# Modeling Reduced Contractility and Stiffness Using iPSC-Derived Cardiomyocytes Generated From Female Becker Muscular Dystrophy Carrier

**DOI:** 10.1016/j.jacbts.2022.11.007

**Published:** 2023-02-08

**Authors:** Satoshi Kameda, Shuichiro Higo, Mikio Shiba, Takumi Kondo, Junjun Li, Li Liu, Tomoka Tabata, Hiroyuki Inoue, Shota Okuno, Shou Ogawa, Yuki Kuramoto, Hideki Yasutake, Jong-Kook Lee, Seiji Takashima, Yoshihiko Ikeda, Shungo Hikoso, Shigeru Miyagawa, Yasushi Sakata

**Affiliations:** a Department of Cardiovascular Medicine, Osaka University Graduate School of Medicine, Suita, Osaka, Japan; b Department of Medical Therapeutics for Heart Failure, Osaka University Graduate School of Medicine, Suita, Osaka, Japan; c Cardiovascular Division, Osaka Police Hospital, Tennoji, Osaka, Japan; d Department of Cardiovascular Surgery, Osaka University Graduate School of Medicine, Suita, Osaka, Japan; e Department of Design for Tissue Regeneration, Osaka University Graduate School of Medicine, Suita, Osaka, Japan; f Department of Cardiovascular Regenerative Medicine, Osaka University Graduate School of Medicine, Suita, Osaka, Japan; g Department of Medical Biochemistry, Osaka University Graduate School of Medicine, Suita, Osaka, Japan; h Department of Pathology, National Cerebral and Cardiovascular Center, Suita, Osaka, Japan

**Keywords:** Becker muscular dystrophy, collagen, *DMD*, human induced pluripotent stem cell–derived cardiomyocytes, *PLOD3*

## Abstract

Study investigators encountered a female Becker muscular dystrophy (BMD) carrier with advanced heart failure (HF) and identified a stop-gain variant in procollagen-lysine, 2-oxoglutarate 5-dioxygenase 3 (*PLOD3*) as a potential second-hit variant. Isogenic induced pluripotent stem cells (iPSCs) with dominant expression of WT-*DMD*, Δ45-48-*DMD*, or Δ45-48-*DMD* with corrected *PLOD3* variant were established. Microforce testing using 3-dimensional self-organized tissue rings (SOTRs) generated from iPSC-derived cardiomyocytes (iPSC-CMs) demonstrated that correction of the heterozygous *PLOD3* variant did not improve the reduced force, but it significantly recovered the reduced stiffness in Δ45-48-*DMD* SOTRs. Correction of the *PLOD3* variant restored collagen synthesis in iPSC-CMs. Our findings revealed the pathogenesis underlying advanced HF in a female BMD carrier.

## Highlights

• Study investigators encountered a rare case of a female BMD carrier with advanced HF who exhibited expression of a Δ45-48 dystrophin in approximately 40% of her heart tissue.
• A stop-gain variant in *PLOD3* was identified as a potential second-hit variant in the proband, which was not detected in her nonmanifesting sister.
• Isogenic iPSCs with dominant expression of WT-*DMD*, Δ45-48-*DMD*, or Δ45-48-*DMD* with a corrected *PLOD3* variant were established using X-chromosome inactivation and genome editing.
• Microforce testing using 3-dimensional SOTRs demonstrated that correction of the heterozygous *PLOD3* variant did not improve the reduced force, but it significantly recovered the reduced stiffness in Δ45-48-*DMD* SOTRs.
• Correction of the heterozygous *PLOD3* variant restored collagen synthesis in iPSC-CMs.

## Visual Abstract

**Figure:**
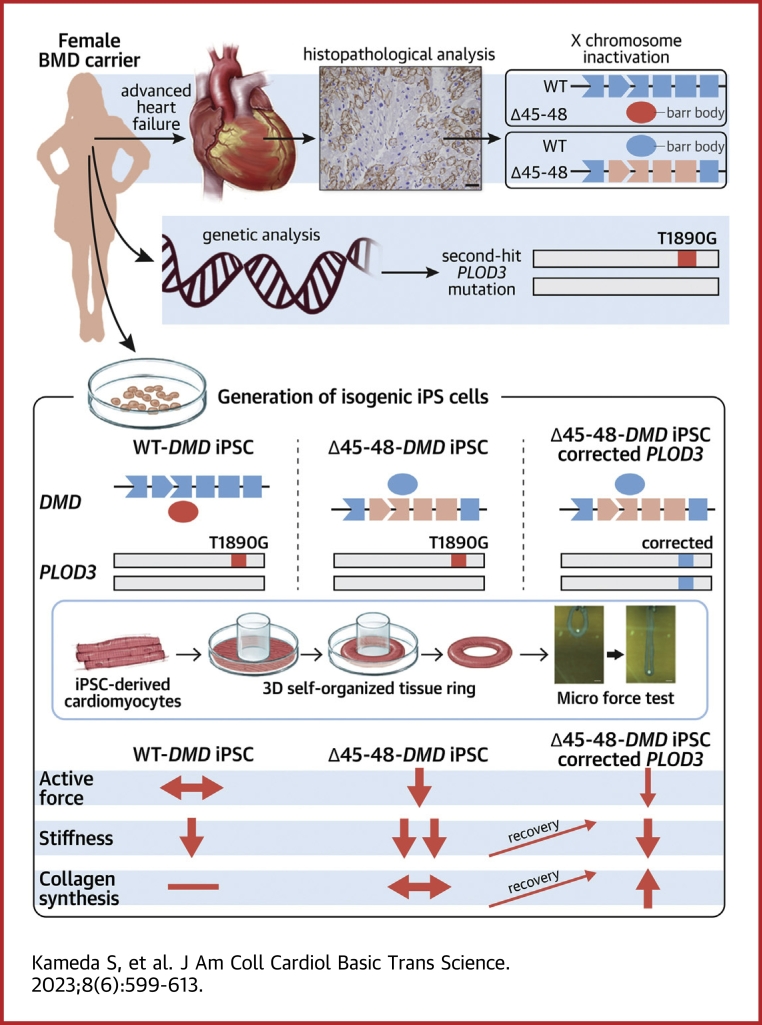

Duchenne muscular dystrophy (DMD) and Becker muscular dystrophy (BMD) are X-linked genetic disorders caused by variants in the *DMD* gene, which encodes the dystrophin protein and is required for structural stability of the sarcolemma.^1^ The prevalence of DMD and BMD in boys and men is 4.78 and 1.53 per 100,000, respectively.^2^ DMD is caused by the absence of dystrophin expression secondary to an out-of-frame deletion within *DMD*. In contrast, patients with BMD have a preserved dystrophin reading frame and thus exhibit a milder form of the disease with a later onset and much longer survival compared with patients with DMD.^3^ The life expectancy for patients with DMD is reported to be 22 to 28 years,^4^ whereas patients with BMD may live until the sixth decade of life.^5^ Cardiac involvement in male patients with BMD is common,^6^ with a frequency of 60% to 75% and an average age of cardiac involvement onset of 28.7 years.^5^ On the contrary, the prevalence of dilated cardiomyopathy in female BMD/DMD carriers is lower compared with that in male patients and ranges from 7% to 18%.^7^^,^^8^ A follow-up study on female BMD/DMD carriers reported that only 1 BMD carrier and 10 DMD carriers of a total of 99 DMD/BMD carriers developed dilated cardiomyopathy (DCM) during a 9-year follow-up period, and none of BMD carriers died.^9^ Meanwhile, a cross-sectional study of 130 female BMD/DMD carriers found that the mean left ventricular (LV) ejection fraction was 63%, and only 4 carriers developed contractile dysfunction with a reduced ejection fraction of <40% at ages 30 to 60 years.^10^ Furthermore, a study of a cohort of 397 female BMD/DMD carriers revealed that the clinical feature of cardiomyopathy is not associated with a reduced life expectancy or an increased risk of cardiac death.^11^ These clinical studies suggest that early onset DCM with advanced HF in female BMD carriers is extremely rare.

During the past decade, numerous studies using induced pluripotent stem cell (iPSC)–derived cardiomyocytes (iPSC-CMs) for modeling BMD/DMD pathogenesis have been reported.^12^ More recently, iPSCs have been generated from male patients with BMD^13^^,^^14^; however, there have been no reports of experimental studies using iPSC-CMs generated from female BMD carriers. Functional analysis of female carriers of BMD using iPSC-CMs is challenging because iPSCs generated from girls and women carry 2 X chromosomes, 1 of which undergoes X chromosome inactivation (XCI).^15^ Furthermore, large exon deletions in *DMD* cannot be corrected by conventional genome editing, thus making it difficult to generate isogenic iPSCs.

In the current study, we encountered a young female BMD carrier with an in-frame deletion in *DMD* of exon 45-48 (Δ45-48) who developed early onset DCM with severe advanced HF that required implantation of an LV assist device (LVAD).^16^ We determined the proportion of the XCI state in the proband’s LV myocardium and identified a potential second-hit variant in the proband that was not present in her nonmanifesting sister. To determine the pathologic effect of the genetic variants, we also performed functional analysis using self-organized tissue rings (SOTRs) generated from isogenic iPSC-CMs prepared using the XCI state and genome editing. Our analysis using isogenic iPSC-CMs revealed the pathogenesis underlying advanced HF in a female BMD carrier.

## Methods

### Human sample

The use of human samples and the genomic analysis were approved by the Ethics Committee of Osaka University Hospital in Osaka, Japan. All investigations were conformed to Ethical Guidelines for Medical and Health Research Involving Human Subjects in Japan and to all principles outlined by the Declaration of Helsinki. We obtained informed consent from the proband and her family.

### Transfection of plasmids into human iPSCs and selection of the targeted clones

Plasmid constructs for genome editing were transfected into iPSCs as described previously.^17^^,^^18^

### Generation of self-organized tissue rings

The 3-dimensional SOTRs were generated as described previously.^19^^,^^20^

### Statistical analysis

Statistical analysis was performed using JMP software version 14.3.0 (SAS Institute Inc). Graphs were prepared using GraphPad Prism software version 9.1.0 (GraphPad Software, Inc). The data were analyzed using the Shapiro-Wilk test to examine normal distribution. Student’s *t*-test was used to compare 2 groups with continuous variables that were normally distributed. Normally distributed data in more than 3 groups were analyzed by analysis of variance, followed by Bonferroni’s post hoc test for multiple pairwise comparisons. Data that were not normally distributed were analyzed by the Mann-Whitney test for comparison in 2 groups or the Kruskal-Wallis test followed by a post hoc test (Steel-Dwass test) for comparison in more than 3 groups. All data are expressed as mean ± SD (normally distributed) or median with 25th and 75th percentiles (not normally distributed). Statistical significance was set at *P* < 0.05.

Expanded experimental methods are provided in the Supplemental Appendix (Supplemental Methods, Supplemental Table 1).

## Results

### A young female BMD carrier with advanced HF exhibited an approximately 6:4 expression ratio of wild-type (WT) dystrophin to Δ45-48 dystrophin in heart tissue

**Figure 1:**
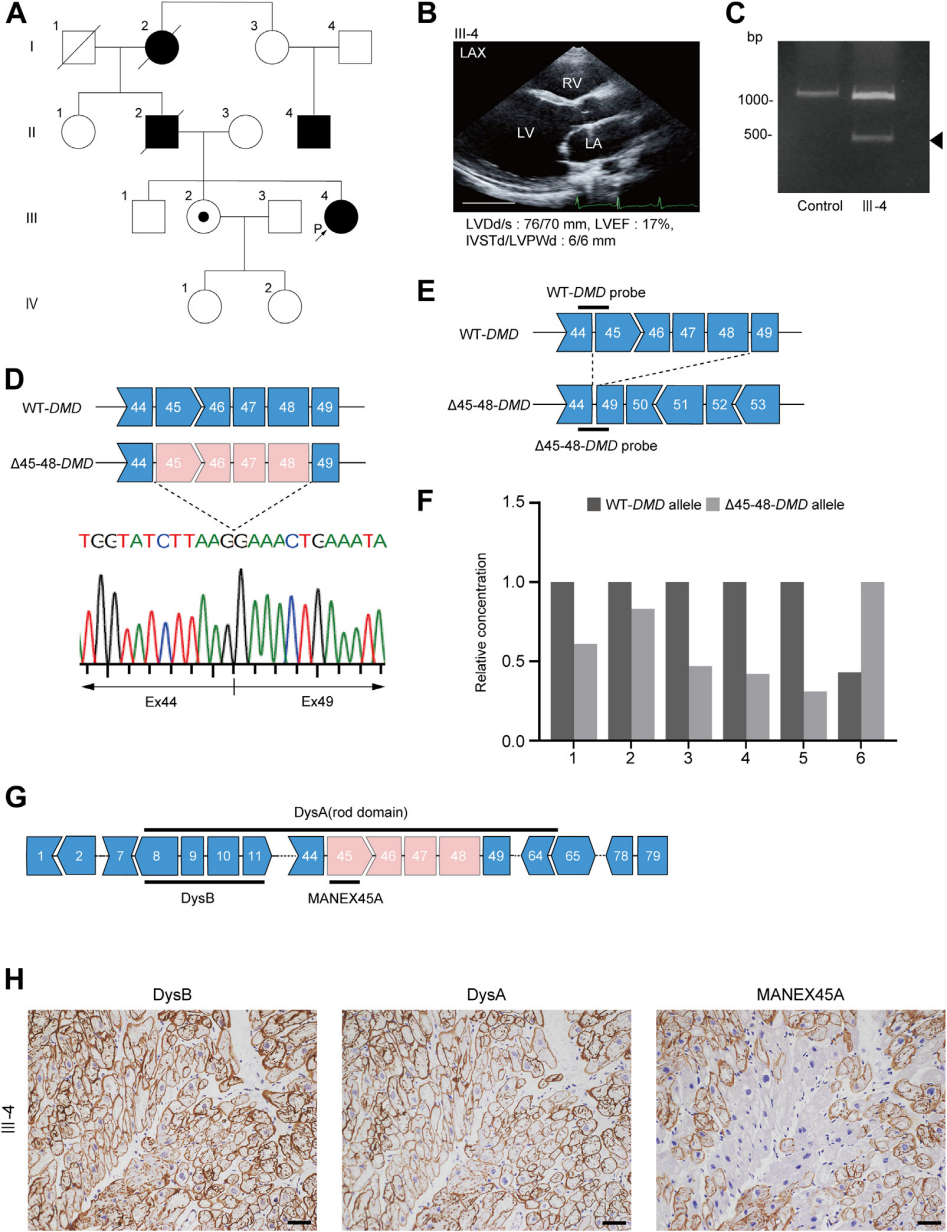
Differential Expression Analysis of WT and Δ45-48 Dystrophin in Heart Tissue of a Female Becker Muscular Dystrophy Carrier With Advanced Heart Failure **(A)** Family pedigree chart of the proband. Cases who presented with heart failure or muscular dystrophy are shown as **black circles** (female) or **black boxes** (male). Deletion of exons (Ex) 45 to 48 (Δ45-48) in *DMD* gene was identified in the proband (III-4) as indicated by an **arrow** and in her nonmanifesting older sister (III-2) as indicated by a **white circle with a dot**. **(B)** Echocardiographic finding of the proband at 29 years of age. (Scale bar, 50 mm.) **(C)** Polymerase chain reaction analysis using cDNA of left ventricular tissues obtained from the proband and a dilated cardiomyopathy control. The polymerase chain reaction product of the proband exhibited 2 bands (1,075 bp and 415 bp). **(D)** Results of direct Sanger sequence analysis using the lower band **(black arrow in C)** demonstrate the deletion between exons 45 and 48 in *DMD*. **(E)** Schematic of the droplet digital polymerase chain reaction probes. The wild-type (WT) probe was designed across exons 44 and 45, whereas the Δ45-48 probe was designed across exons 44 and 49. **(F)** Results of the droplet digital polymerase chain reaction analysis. The concentration (copies/μL) of each transcript in the cDNA samples was normalized to that of the dominant transcript. **(G)** Location of the epitopes in dystrophin targeted by each antibody: DysA, DysB, and MANEX45A (Developmental Studies Hybridoma Bank). **(H)** Immunohistochemical analysis of the left ventricular myocardium obtain from the proband using the indicated antibodies. (Scale bars, 50 μm.) IVSTd = interventricular septal thickness at end-diastole; LA = left atrium; LAX = left parasternal long axis view; LV = left ventricle; LVDd = left ventricular diastolic diameter; LVDs = left ventricular systolic diameter; LVEF = left ventricular ejection fraction; LVPWd = left ventricular posterior wall thickness at end-diastole; RA = right atrium; RV = right ventricle.

We encountered a rare case of a 29-year-old female BMD carrier (proband, Ⅲ-4) (Figure 1A) with rapidly progressive severe HF who underwent LVAD implantation.^16^ Echocardiography at 29 years of age revealed a visibly enlarged left ventricle and severe diffuse LV hypokinesis with an ejection fraction of 17% (Figure 1B). In the family history, her paternal grandmother (Ⅰ-2) died of HF in her 80s, and a paternal relative (Ⅱ-4) had a diagnosis of muscular dystrophy. Her father (Ⅱ-2) had received a diagnosis of DCM and died of HF in his 40s. Her mother (Ⅱ-3), older brother (Ⅲ-1), and older sister (Ⅲ-2) have not shown apparent symptoms of heart disease or muscular dystrophy according to the available clinical records. Multiplex ligation-dependent probe amplification using peripheral blood samples identified a heterozygous Δ45-48 in *DMD* of the proband and her older sister (Ⅲ-2). On the basis of the clinical course and genetic analysis, the proband received a diagnosis of a BMD carrier with DCM. Her older sister (Ⅲ-2) received a diagnosis of a nonmanifesting BMD carrier.

XCI is responsible for sex chromosome dosage compensation in girls and women.^15^ Analysis of genomic DNA extracted from peripheral blood demonstrated that a skewed XCI is associated with cardiac dysfunction in DMD carriers^21^ and BMD^22^ carriers. However, the proportion of XCI in heart tissue of BMD carriers has not been determined yet. Polymerase chain reaction (PCR) and Sanger sequence analysis using complementary DNA (cDNA) prepared using RNA isolated from the LV heart tissue of the proband confirmed the Δ45-48 variant in *DMD* (Figures 1C and 1D). Specific probes were designed to evaluate the differential expression of WT and Δ45-48 transcripts from *DMD* (Figure 1E). Droplet digital PCR (ddPCR) analysis using cDNA obtained from the proband’s LV tissues (n = 6) revealed that the average ratios of WT and Δ45-48 transcripts were 59.3% and 40.7%, respectively (Figure 1F). To evaluate the differential expression of WT and Δ45-48 dystrophin proteins further, immunohistochemical analysis of the proband’s LV myocardium was performed using 3 different antibodies: 1) DysA against the rod domain^23^; 2) DysB against the N-terminal domain^24^; and 3) MANEX45A (Developmental Studies Hybridoma Bank) against the domain derived from exon 45 of the dystrophin protein (Figure 1G).^25^ These 3 antibodies uniformly detected the expression of dystrophin in the LV heart tissue of a patient with no history of cardiac disease (Supplemental Figure 1A). Immunohistochemical analysis of serial sections obtained from the proband’s LV myocardium demonstrated that the DysA antibody detected patchy and faint expression of dystrophin compared with that of the DysB antibody (Figure 1H). Immunostaining using the MANEX45A antibody specifically detected WT dystrophin expression. Quantitative analysis determined that the ratios of WT and Δ45-48 dystrophins were approximately 59.0% and 41.0%, respectively, findings consistent with the result from the ddPCR analysis. These data suggested an approximately 6:4 expression ratio of WT dystrophin to that of Δ45-48 dystrophin in the heart tissue of the proband with advanced HF.

### Lysyl hydroxylase 3 (LH3) levels decreased in the heart tissue of the proband in response to a heterozygous stop-gain variant

**Figure 2:**
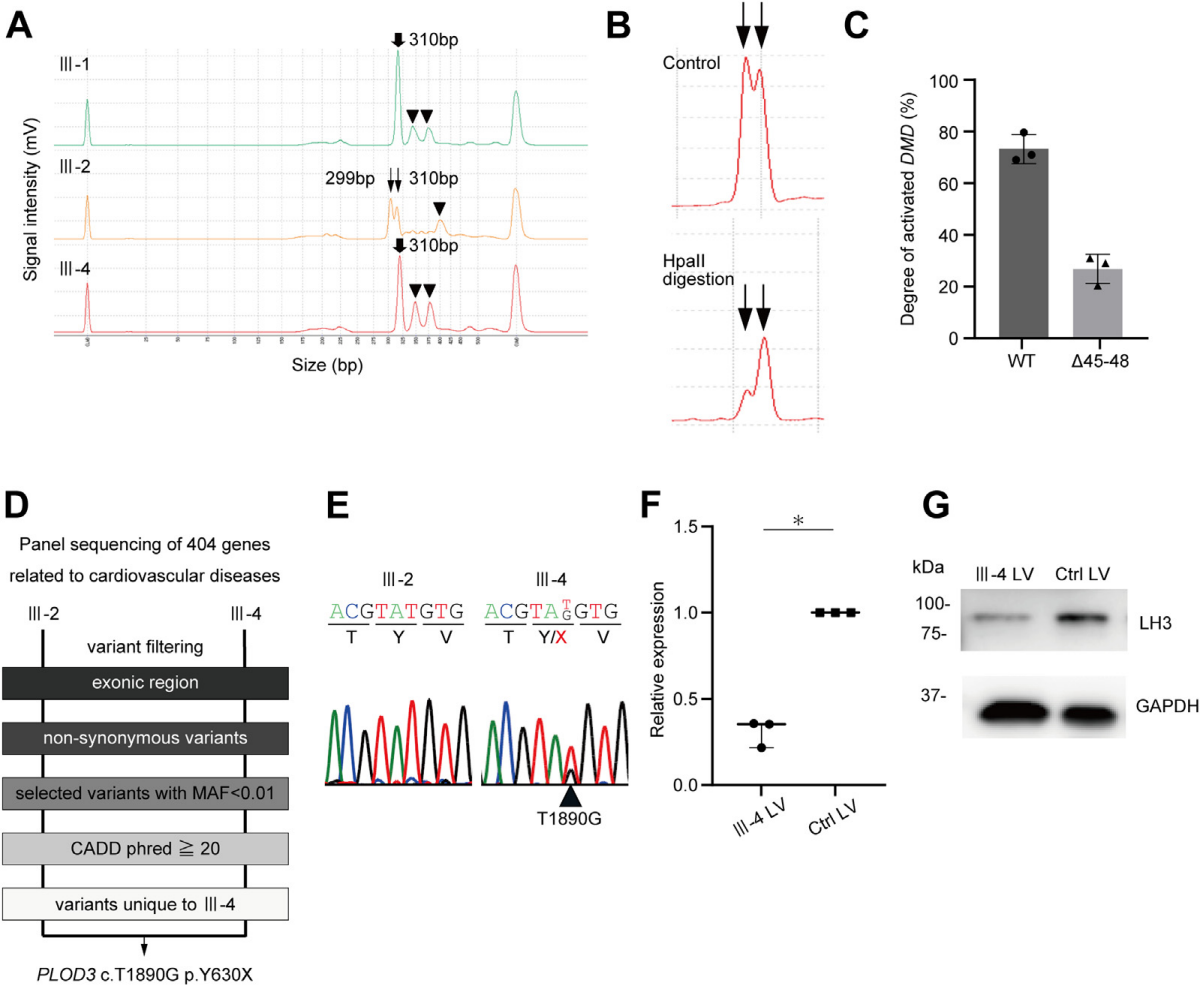
X Chromosome Inactivation Assay and Identification of the *PLOD3* Variant **(A)** The polymerase chain reaction product generated from amplification of genomic DNA for each case was analyzed using a microchip electrophoresis system (MultiNA, Shimadzu). The polymerase chain reaction products from the proband (Ⅲ-4) and her older brother (Ⅲ-1) showed single peaks (**bold arrows**, 310 bp), whereas the proband’s older sister (Ⅲ-2) showed bimodal peaks (**narrow arrows**, 299 bp and 310 bp). The higher-molecular-weight signals **(arrowheads)** are believed to be oligomers of polymerase chain reaction products. **(B)** Representative result of the X chromosome inactivation assay before (control, **upper**) and after (HpaⅡ digestion, **lower**) enzyme digestion. The lower-molecular-weight band derived from the wild-type (WT) allele was more prone to digestion than that of the higher-molecular-weight band, which was derived from the deletion of exons 45 to 48 (Δ45-48) allele. **(C)** The degree of activated WT or Δ45-48-*DMD* gene in the X chromosome of the proband’s older sister (Ⅲ-2) was calculated from the peak area before and after enzyme digestion. The calculated average degree of activated WT and Δ45-48-*DMD* gene was 73.2% and 26.8%, respectively (n = 3). **(D)** Flow chart of the filtering process for the identified of exonic variants. **(E)** Sanger sequence analysis confirmed the heterozygous procollagen-lysine, 2-oxoglutarate 5-dioxygenase 3 *(PLOD3)* T1890G variant in the proband (Ⅲ-4). **(F)** Relative expression of *PLOD3* normalized by the glyceraldehyde 3-phosphate dehydrogenase (*GAPDH)* gene in the left ventricular heart tissues of the proband (Ⅲ-4) (n = 3) and control (Ctrl) dilated cardiomyopathy (n = 3) were measured using quantitative polymerase chain reaction analysis. Relative expression levels normalized to the control are shown. ∗*P* < 0.05. **(G)** Whole-cell lysates were extracted from left ventricular tissue of the proband (Ⅲ-4) and control dilated cardiomyopathy and were analyzed by Western blotting using the indicated antibodies. CADD = combined annotation-dependent depletion; LH3 = lysyl hydroxylase 3; LV = left ventricle; MAF = minor allele frequency.

Substantial expression of Δ45-48 *DMD* in the heart tissue may have accounted for the rapid progression of the cardiac dysfunction observed in the proband, but this was not observed in her older sister (Ⅲ-2), whose cardiac function was within normal limits (Supplemental Figure 1B). To compare the XCI states between the sisters, we performed an androgen receptor *(AR)* methylation-based assay using peripheral blood,^21^^,^^22^ given that a myocardium sample of the Ⅲ-2 carrier was unavailable. Analysis of the XCI state in girls and women by using blood samples is considered comparable with that of other tissues that may be inaccessible.^26^ XCI detection using methylation-based assays to distinguish XCI states is dependent on the CAG repeat polymorphism in each X chromosome; therefore, 2 different peaks must be detected during the electrophoretic analysis of PCR products spanning the CAG repeats of the *AR* gene. As expected, a single peak was detected in the proband’s older brother (Ⅲ-1) who was carrying only 1 X chromosome (Figure 2A). Only 1 peak was also detected in the proband (Ⅲ-4), a finding suggesting that the number of CAG repeats in the 2 X chromosomes was comparable. However, 2 peaks were detected in the Ⅲ-2 carrier, with the lower-molecular-weight peak estimated to be derived from the WT-*DMD* allele, because Ⅲ-2 and Ⅲ-4 (sisters) inherited a common X chromosome carrying the Δ45-48-*DMD* allele. Digestion with endonuclease HpaII and quantitative analysis revealed that the proportion of WT-*DMD* expression to Δ45-48-*DMD* expression was approximately 7:3 (Figures 2B and 2C). This suggested that substantial Δ45-48-*DMD* expression in heart tissue would also be predicted in the nonmanifesting carrier Ⅲ-2. These findings prompted us to explore another contributing factor for advanced HF in the proband. We screened the expression of a total of 404 different genes related to inherited cardiovascular disease in the proband (Ⅲ-4) and her sister (Ⅲ-2),^27^ and we identified a heterozygous stop-gain variant (c.T1890G p.Y630X) in procollagen-lysine, 2-oxoglutarate 5-dioxygenase 3 (*PLOD3*), which encodes LH3 and was detected only in the proband (Figures 2D and 2E). LH3 is ubiquitously expressed in various organs, including the heart, and it catalyzes the hydroxylation of lysyl residues of collagens and plays an important role in collagen cross-linking and deposition.^28^, ^29^, ^30^ Compound heterozygous variants or homozygous variants in *PLOD3* of humans results in a connective tissue disorder.^29^^,^^31^^,^^32^ Both the mRNA expression levels of *PLOD3* and the protein expression levels of LH3 were decreased in the proband’s LV tissue compared with that of the control DCM patient (Figures 2F and 2G).

### Generation of isogenic iPSCs using the XCI state and genome editing

**Figure 3:**
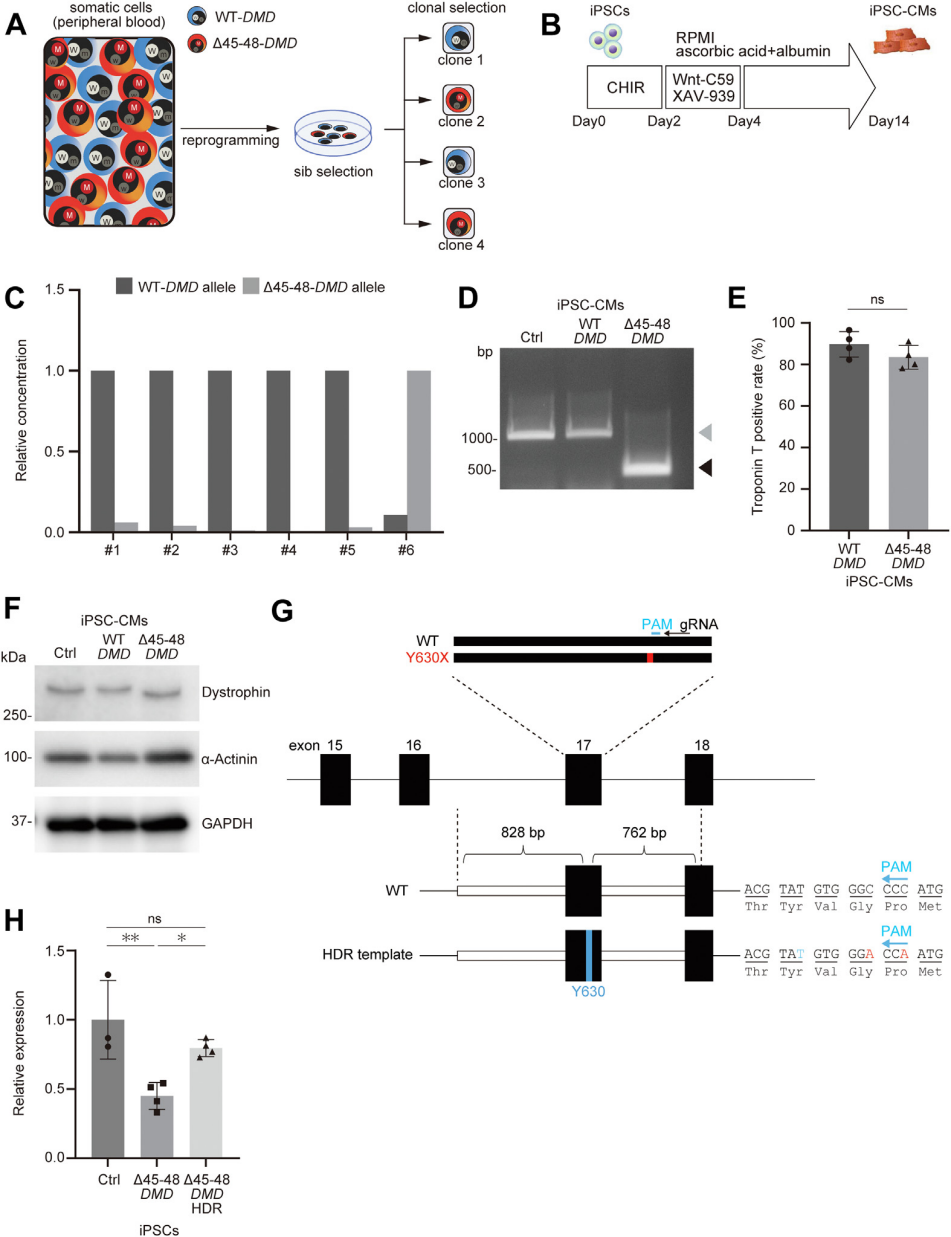
Generation and Characterization of the Isogenic iPSCs **(A)** Schematic of the establishment of induced pluripotent stem cells (iPSCs) from peripheral blood of the proband and the selection of the induced pluripotent stem cell clone with dominant expression of wild-type (WT) or deletion of exons 45 to 48 (Δ45-48)-*DMD* gene. **(B)** Time course for the differentiation into cardiomyocytes. After differentiation, induced pluripotent stem cell–derived cardiomyocytes (iPSC-CMs) were used for droplet digital polymerase chain reaction analysis. **(C)** Results of droplet digital polymerase chain reaction analysis using cDNA samples obtained from 6 different clones of patient-derived induced pluripotent stem cell–derived cardiomyocytes. The concentration (copies/μL) of each transcript was normalized to that of the dominant transcript. **(D)** Polymerase chain reaction analysis using cDNA obtained from WT-*DMD* and Δ45–48-*DMD* induced pluripotent stem cell–derived cardiomyocytes. The amplicon size of the control and WT-*DMD* induced pluripotent stem cell–derived cardiomyocytes is 1,075 bp **(gray arrowhead)**, and that of Δ45–48-*DMD* induced pluripotent stem cell–derived cardiomyocytes is 415 bp **(black arrowhead)**. **(E)** Troponin T–positive rate of WT-*DMD* (n = 4) and Δ45–48-*DMD* induced pluripotent stem cell–derived cardiomyocytes (n = 4) determined by fluorescence-activated cell sorting. **(F)** Western blot analysis of total protein extracts from control, WT-*DMD*, and Δ45–48-*DMD* induced pluripotent stem cell–derived cardiomyocytes using the indicated antibodies. **(G)** Schematic of genome editing of procollagen-lysine, 2-oxoglutarate 5-dioxygenase 3 (*PLOD3)* in Δ45–48-*DMD* induced pluripotent stem cells. The guide RNA (gRNA) targets a common sequence downstream of heterozygous Y630X. The homology-directed repair (HDR) repair template consists of a 5-terminal 828-bp and a 3-terminal 762-bp homology arm corresponding to the genomic sequence proximal to exon 17 of *PLOD3*. To avoid recleavage by Cas9, synonymous variants were introduced **(red).** T1890 is highlighted in **blue**. **(H)** Relative expression levels of *PLOD3* normalized to the glyceraldehyde 3-phosphate dehydrogenase *(GAPDH)* gene were measured using quantitative polymerase chain reaction in control (Ctrl) (n = 3), Δ45–48-*DMD* (n = 4), and Δ45–48-*DMD*-homology-directed repair induced pluripotent stem cells (n = 4). Relative expression levels normalized to the control are shown. ∗*P* < 0.05; ∗∗*P* < 0.01. PAM = protospacer adjacent motif.

To determine the functional consequence of variants in *DMD* and *PLOD3*, we sought to establish isogenic iPSC-CMs in disease modeling. We generated iPSCs from mononuclear leukocytes obtained from the peripheral blood of the proband. Inactivation of maternal or paternal X chromosomes in somatic cells occurs randomly.^15^^,^^33^ Therefore, iPSCs generated from somatic cells derived from the proband’s peripheral blood were expected to express either WT or Δ45-48-*DMD* transcripts (Figure 3A). Because *DMD* is expressed only in differentiated cardiomyocytes, and not in iPSCs, cardiomyocyte differentiation was induced using a chemically defined protocol (Figure 3B).^34^ A total of 6 clones were then screened using ddPCR analysis. Among them, clone #1 was identified to exhibit dominant expression of WT-*DMD* (WT-*DMD* iPSC), whereas clone #6 exhibited dominant expression of Δ45-48-*DMD* (Δ45-48-*DMD* iPSC) (Figures 3C and 3D). Both clone #1 and clone #6 were positive for SSEA4, TRA-1–60, OCT3/4, and NANOG, and they had normal karyotypes (Supplemental Figures 2A and 2B). Differentiation efficiencies of WT-*DMD* and Δ45-48-*DMD* iPSC-CMs were determined by fluorescence-activated cell sorting analysis using an anti–troponin T antibody and were found to be comparable, with more than 80% efficiency on day 14 post-differentiation (Figure 3E). Western blot analysis detected the full-length dystrophin protein and the shortened dystrophin protein in WT-*DMD* and Δ45-48-*DMD* iPSC-CMs, respectively (Figure 3F). Reactivation of the inactive X-linked genes (Xi) to biallelic expression, a process called Xi erosion, is known to progress with time in stem cells in culture.^35^, ^36^, ^37^ We investigated whether Xi erosion affected *DMD* expression from the inactivated allele in the WT-*DMD* and Δ45-48-*DMD* iPSC-CMs. Using ddPCR analysis of iPSC-CMs that were differentiated from serially passaged iPSCs, we found that Δ45-48 or WT-*DMD* transcripts initially repressed were subsequently expressed in both WT-*DMD* and Δ45-48-*DMD* iPSC-CMs after 30 passages (Supplemental Figure 2C). On the basis of these results, we performed additional experiments using iPSCs that were cultured for fewer than 20 passages to avoid any effect from Xi erosion. We also sought to repair the heterozygous Y630X variant in *PLOD3* in Δ45-48-*DMD* iPSCs by genome editing using guide RNA (gRNA) and repair template DNA containing a synonymous variant to avoid Cas9-mediated recleavage (Figure 3G). Through homology-directed repair (HDR), a homozygous corrected clone was obtained (Δ45–48-*DMD*-HDR iPSCs) in which both *PLOD3* alleles were replaced with the synonymous variant (Supplemental Figures 3A and 3B). The expression levels of *PLOD3* mRNA in Δ45-48-*DMD*-HDR iPSCs were recovered from the Δ45-48-*DMD* iPSCs to levels comparable with those in iPSCs generated from a female healthy subject (control iPSCs) (Figure 3H).

### Correction of the heterozygous *PLOD3* stop-gain variant rescued the decreased stiffness

**Figure 4:**
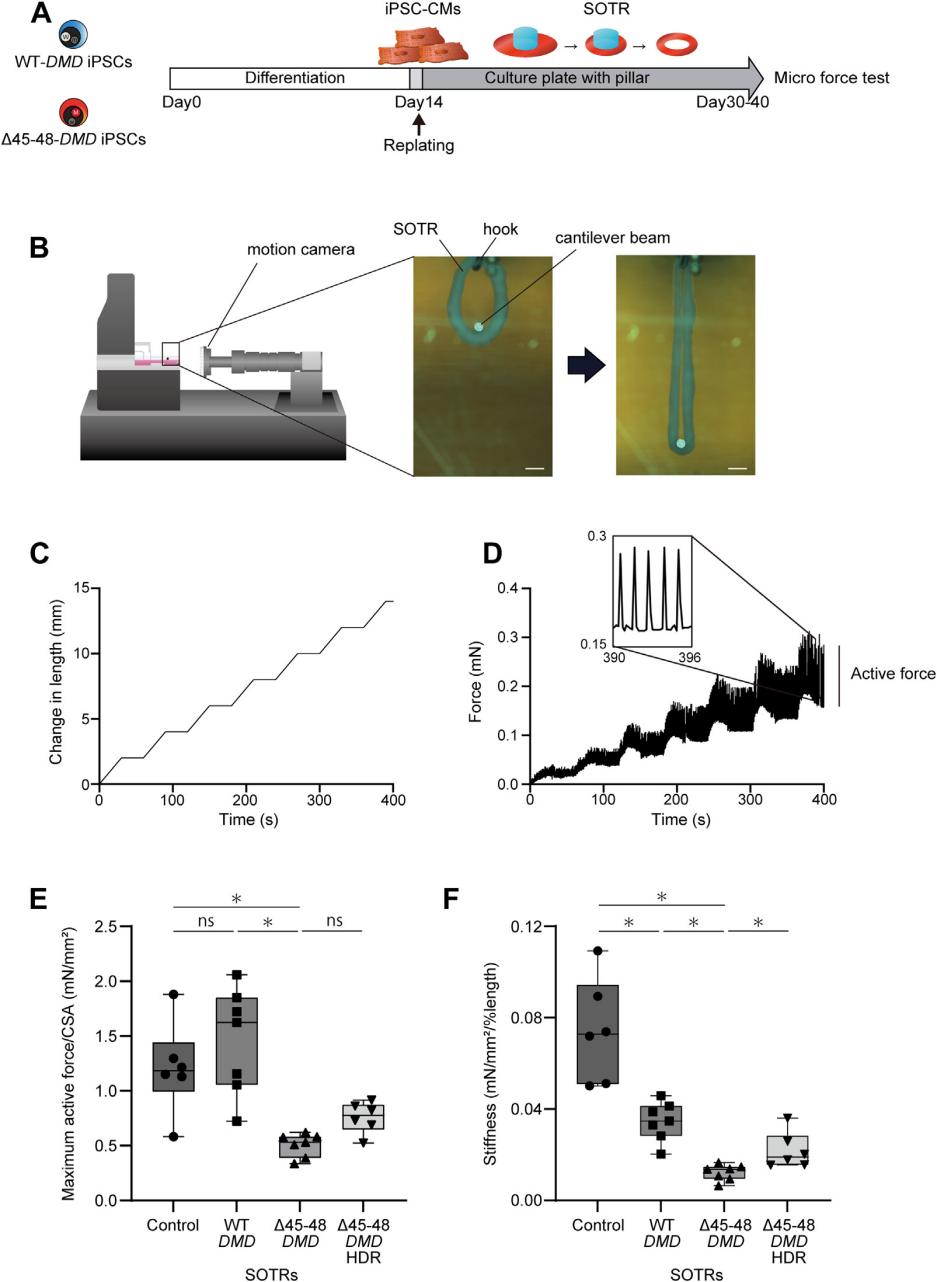
Microforce Analysis Using SOTRs Generated From Isogenic iPSC-CMs **(A)** Time course of self-organized tissue ring (SOTR) generation and Microforce testing. **(B)** The force was calculated on the basis of the beam deflection in response to differential displacement under increasing stretch. Micromotion of the beam and self-organized tissue ring were detected using a motion camera located in front of the samples. (Scale bars, 600 μm.) **(C)** Stepwise stretch at 30-second intervals on self-organized tissue rings. **(D)** Active force measurement under the stepwise stretch. **(E)** Maximum active force corrected by cross-sectional area (CSA) was compared among control (n = 6), wild-type (WT)- *DMD* gene (n = 7), deletion of exons 45 to 48 (Δ45–48)-*DMD* (n = 7), and Δ45–48-*DMD*-homology-directed repair (HDR) self-organized tissue rings (n = 6). ∗*P* < 0.05. **(F)** Stiffness was compared among control (n = 6), WT-*DMD* (n = 7), Δ45–48-*DMD* (n = 7), and Δ45–48-*DMD*-homology-directed repair self-organized tissue rings (n = 6). ∗*P* < 0.05. iPSC-CM = induced pluripotent stem cell–derived cardiomyocyte.

There are reports of the establishment of iPSCs derived from patients with BMD^13^^,^^14^^,^^38^; however, the functional properties of cardiomyocytes in engineered tissues under isogenic backgrounds have not been studied. Therefore, we generated 3-dimensional SOTRs from WT-*DMD*, Δ45-48-*DMD*, Δ45-48-*DMD*-HDR, and control iPSC-CMs differentiated from control iPSCs generated from a healthy subject (WT-*DMD*, Δ45-48-*DMD*, Δ45-48-*DMD*-HDR, and control SOTRs, respectively) and evaluated their contractile force and stiffness by using a MicroTester G2 microscale mechanical test system (CellScale Biomaterials Testing) as described previously.^19^^,^^20^ Differentiated iPSC-CMs were seeded into 24-well plates with central pillars on day 14 post-differentiation that then spontaneously formed SOTRs within 7 days (Figure 4A). The generated SOTRs were removed from the pillar at 30 to 40 days post-differentiation and were hung on the MicroTester G2 hook (Figure 4B). The SOTRs were attached by a cantilever beam, stretched 2 mm in 30 seconds, and then held for 30 seconds for 1 cycle (Figure 4C). Transient active force was detected under these conditions as twitch force amplitude,^39^^,^^40^ which increased with increased stretches (Figure 4D). The passive stiffness was calculated as the slope of the diastolic force vs the length ratio at the first 25% length (Supplemental Figure 3C), which is considered to be a physiologically relevant parameter.^39^^,^^40^ The active force per cross-sectional area of WT-*DMD* SOTRs was comparable with that of control SOTRs (1.456 ± 0.484 and 1.209 ± 0.415 mN/mm^2^; *P* = 0.92) (Figure 4E). The active force of Δ45-48-*DMD* SOTRs was significantly decreased compared with that of WT-*DMD* SOTRs (0.507 ± 0.106 mN/mm^2^; *P* = 0.012), and correction of the heterozygous *PLOD3* stop-gain variant in Δ45-48-*DMD*-HDR SOTRs showed a trend but did not significantly improve the reduced force (0.757 ± 0.142 mN/mm^2^; *P* = 0.086). The stiffness was significantly decreased in WT-*DMD* SOTRs compared with that of the control SOTRs (0.0346 ± 0.0085 and 0.0743 ± 0.0227 mN/mm^2^/% length; *P* = 0.018) (Figure 4F), and it further decreased in Δ45-48-*DMD* SOTRs (0.0122 ± 0.0035 mN/mm^2^/% length; *P* = 0.012), findings suggesting that haploinsufficiency of *PLOD3* significantly affected the stiffness of SOTRs and that the combined variant of *DMD* with *PLOD3* further exacerbated the phenotype. Of note, the stiffness was significantly recovered in Δ45-48-*DMD*-HDR SOTRs compared with Δ45-48-*DMD* SOTRs (0.0218 ± 0.0080 mN/mm^2^/% length; *P* = 0.041).

### Correction of the heterozygous *PLOD3* stop-gain variant restored collagen synthesis in iPSC-CMs

**Figure 5:**
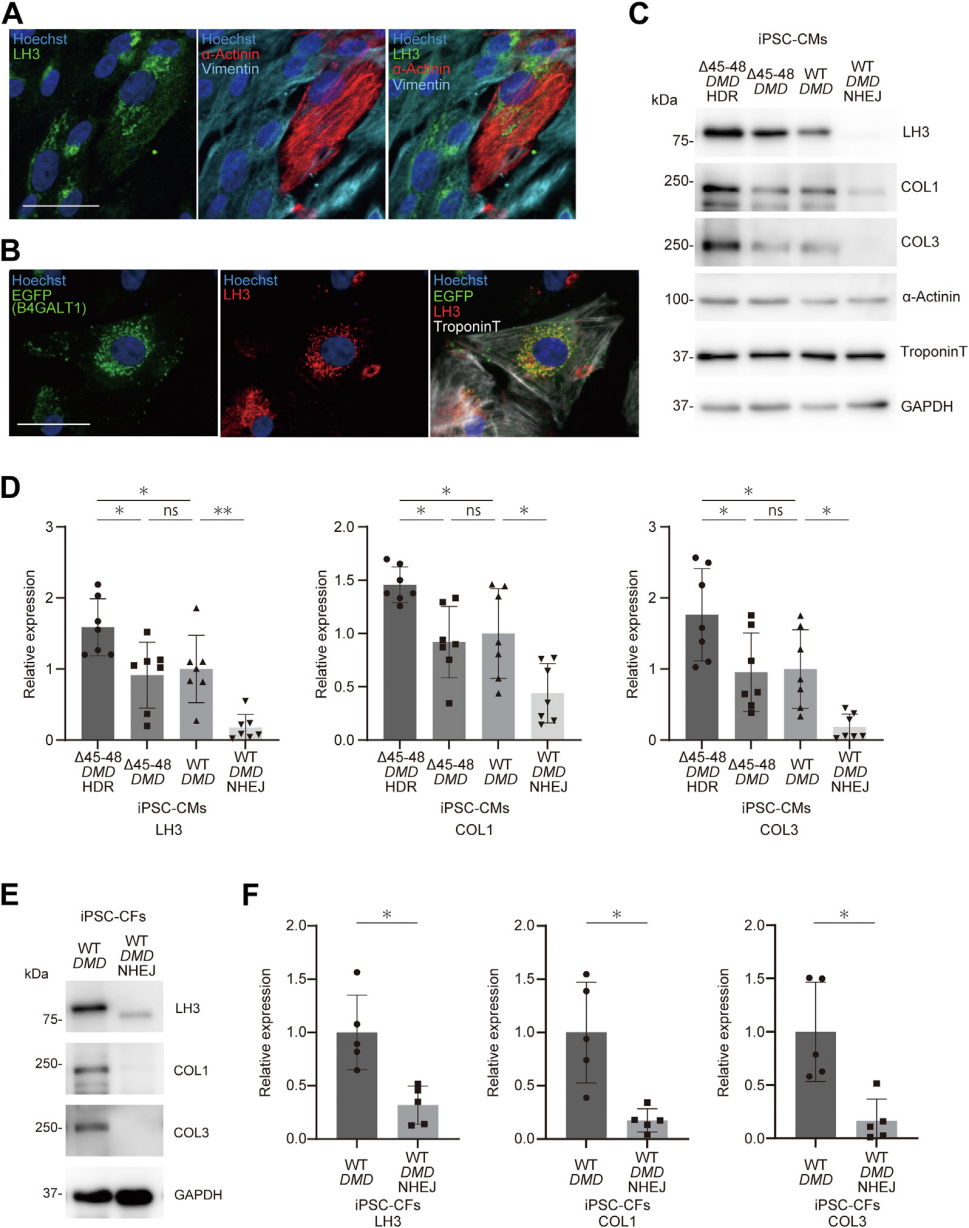
LH3 Expression and Collagen Synthesis in iPSC-CMs **(A)** Wild-type (WT)-*DMD* gene induced pluripotent stem cell–derived cardiomyocytes (iPSC-CMs) were fixed in 96-well plates on day 14 post-differentiation and immunostained using the indicated antibodies. (Nuclei were stained with Hoechst; scale bar, 50 μm.) **(B)** WT-*DMD* induced pluripotent stem cell–derived cardiomyocytes were seeded into 96-well plates on day 14 post-differentiation and transduced with AAV encoding *B4GALT1*-EGFP at 1.0 × 10^4^ vg/cell. The cells were then fixed and immunostained on day 21 post-differentiation using the indicated antibodies. (Scale bar, 50 μm.) **(C)** Whole-cell lysates were extracted from Δ45-48-*DMD*-homology-directed repair (HDR), Δ45–48-*DMD*, WT-*DMD,* and WT-*DMD*- nonhomologous end joining (NHEJ) induced pluripotent stem cell–derived cardiomyocytes on day 30 post-differentiation and analyzed by Western blotting using the indicated antibodies. **(D)** Relative expression of each protein was normalized to glyceraldehyde 3-phosphate dehydrogenase (GAPDH) and quantitatively analyzed (lysyl hydroxylase 3 [LH3], n = 7; collagen I [COL1], n = 7; collagen III [COL3], n = 7). Relative expression levels normalized to the levels in WT-*DMD* induced pluripotent stem cell–derived cardiomyocytes are shown. ∗*P* < 0.05; ∗∗*P* < 0.01. **(E)** Whole-cell lysates were extracted from WT-*DMD* and WT-*DMD*- nonhomologous end joining fibroblasts and analyzed by Western blotting using the indicated antibodies. **(F)** Relative expression of each protein was normalized to glyceraldehyde 3-phosphate dehydrogenase and quantitatively analyzed (lysyl hydroxylase 3, n = 5; collagen I, n = 5; collagen III, n = 5). Relative expression levels normalized to the levels in WT-*DMD* induced pluripotent stem cell–derived CFs are shown. ∗*P* < 0.05. AAV = adeno-associated virus; CF = cardiac fibroblast; EGFP = enhanced green fluorescent protein.

Functional data using SOTRs suggested that Δ45-48 dystrophin decreased both the active force and passive stiffness in iPSC-CMs, with haploinsufficiency of LH3 further exacerbating the stiffness. LH3 catalyzes the hydroxylation of lysyl residues of collagens and plays a role in collagen cross-linking and deposition.^28^, ^29^, ^30^^,^^41^ Under our experimental conditions, LH3 was expressed in both iPSC-CMs and noncardiomyocytes that were positive for vimentin (Figure 5A), which is a marker for iPSC-derived fibroblasts,^42^ and localized to the perinuclear Golgi body (Figure 5B), as reported previously.^43^ To determine the functional consequence of *PLOD3* deficiency in iPSC-CMs, we generated an iPSC clone in WT-*DMD* iPSCs by non-homologous end joining (NHEJ) that contained homozygous frameshift variants of *PLOD3* (M634fs687X/Y630X, WT-*DMD*-NHEJ iPSCs) (Supplemental Figures 4A and 4B). The mRNA expression levels of *PLOD3* were significantly decreased in the WT-*DMD*-NHEJ iPSCs compared with those in the WT-*DMD* iPSCs (Supplemental Figure 4C). Importantly, loss of LH3 significantly decreased stiffness in WT-*DMD*-NHEJ SOTRs compared with WT-*DMD* SOTRs, although it did not affect active force (Supplemental Figure 4D). Western blot analysis demonstrated that the protein expression levels of type I and type III collagen were significantly decreased in the WT-*DMD*-NHEJ iPSC-CMs compared with those in the WT-*DMD* iPSC-CMs (Figures 5C and 5D), a finding suggesting LH3 is required for collagen synthesis in iPSC-CMs. Furthermore, type I and type III collagens were significantly decreased in the cardiac fibroblasts differentiated from WT-*DMD*-NHEJ iPSCs compared with those from WT-*DMD* iPSCs (Figures 5E and 5F, Supplemental Figures 5A to 5C), thus supporting the functional role of LH3 in cardiac fibroblasts. Importantly, correction of the heterozygous *PLOD3* stop-gain variant restored both type I and type III collagen levels in Δ45-48-*DMD* iPSC-CMs (Figures 5C and 5D), and the expression levels of collagens in Δ45-48-*DMD*-HDR iPSC-CMs were comparable with those in control iPSC-CMs (Supplemental Figures 5D and 5E). Type I collagen and type III collagen are major components of the cardiac extracellular matrix (ECM),^44^ and the inhibition of collagen cross-linking decreases myocardial stiffness in the left ventricle of pigs.^45^ These findings suggest that the loss of LH3 affects stiffness in iPSC-CMs, which is possibly a result of decreased collagen synthesis.

## Discussion

We encountered a rare case of a young Japanese female BMD carrier with a Δ45-48-*DMD* variant who developed advanced HF with LV systolic dysfunction in her 20s. *DMD* is located on the X chromosome and undergoes epigenetic regulation by XCI, which compensates for the difference in the number of X chromosome genes between male and female subjects.^15^ The nonrandom inactivation of the second X chromosome in somatic cells of girls and women is called skewed XCI and is considered a potential mechanism underlying the clinical symptoms observed in BMD/DMD carriers.^21^^,^^22^ Previous studies using peripheral blood samples provided evidence of XCI in BMD/DMD carriers. However, because most female BMD carriers do not develop severe HF and do not require myocardial biopsy or LVAD implantation, direct evidence of the XCI state in ventricular myocardium is lacking as a result of the unavailability of human heart tissues. In the present study, we precisely evaluated the XCI state in the ventricular myocardium and found that the ratio of WT to Δ45-48 was approximately 6:4 on the basis of immunohistochemical analysis using an exon-specific antibody combined with ddPCR analysis. These findings suggest that the substantial expression of Δ45-48 dystrophin in the heart tissue of the proband may have been a leading cause of reduced cardiac function. However, the XCI states of the proband and her nonmanifesting sister were comparable when evaluated using a methylation-based assay. This result suggested that a varying XCI state may not have accounted for the difference in disease severity observed between the sisters. This prompted us to explore another contributing factor because compound genetic variants may interact to induce DCM.^46^ Genetic analysis targeting 404 different genes involved in inheritable cardiovascular diseases identified a heterozygous stop-gain variant in *PLOD3*, which encodes LH3. LH3 expression was significantly decreased in the proband’s myocardium compared with that in the myocardium of the nonmanifesting sister.

Although the generation of isogenic iPSCs from a female BMD carrier was challenging, we successfully obtained isogenic iPSCs that dominantly expressed either WT or Δ45-48-*DMD* using the random XCI state of source peripheral blood lymphocytes, as previously described.^36^^,^^47^ Furthermore, we established isogenic iPSCs with a correction of the heterozygous *PLOD3* variant. Stepwise stretch analysis using SOTRs generated from the isogenic iPSC-CMs allowed the evaluation of the length-tension relationship according to the Frank-Starling mechanism, which demonstrated that the active force and stiffness were significantly decreased in Δ45-48-*DMD* SOTRs compared with those in WT-*DMD* SOTRs. Mechanical stretch analysis of ex vivo isolated hearts demonstrated that dystrophin-deficient mdx hearts exhibit increased LV compliance, possibly secondary to a perturbed cell-ECM connection and promoted slippage of myocytes.^48^ Our experimental data were consistent with these previous findings, and the human disease model using isogenic SOTRs established in this study may facilitate the pathologic phenotypes resulting from not full-length but truncated dystrophin protein caused by Δ45-48 variant. Microforce testing using isogenic SOTRs further clarified that correction of the heterozygous *PLOD3* variant was able to restore the stiffness in Δ45-48-*DMD* SOTRs. Correction of the heterozygous *PLOD3* variant did not normalize the stiffness to that in control SOTRs, a finding suggesting that the combined variants of Δ45-48-*DMD* and *PLOD3* affected the stiffness in SOTRs.

LH3 is a multifunctional enzyme with lysyl hydroxylase, collagen galactosylysine, and glucosyltransferase activity, and these enzymatic activities are known to be involved in post-translational modifications during collagen biosynthesis.^43^ Loss of *Plod3* in mice disrupts basement membrane structures and leads to embryonic lethality through impaired expression of type Ⅳ and type Ⅵ collagens.^49^^,^^50^ Compound heterozygous or homozygous variants in *PLOD3* cause a systemic connective tissue disorder in humans.^29^^,^^31^^,^^32^ The dystrophin protein links cytoplasmic actin to membrane-localized dystrophin-glycoprotein complexes that connect with the ECM.^51^ On the basis of these findings, we speculate that decreased LH3 expression may impair the recovery process, including collagen synthesis, after myocardial injury resulting from Δ45-48 truncated dystrophin. This may further exacerbate BMD pathogenesis; however, haploinsufficiency of LH3 itself may not suffice to induce a pathologic phenotype.

The main component of the cardiac ECM is collagen, which comprises approximately 85% type Ⅰ collagen and 11% type Ⅲ collagen.^44^ The ratio of type Ⅰ to type Ⅲ collagen increases in the hearts of patients with DCM, and this increased ratio contributes to reduce elasticity of the LV wall and LV dilation.^52^^,^^53^ Type I collagen retains substantial tensile strength, whereas type Ⅲ collagen has the elasticity to maintain the structural integrity of the collagen network.^54^ In particular, increased type Ⅲ collagen expression helps maintain cardiac function in injured myocardium.^53^ Although several environmental factors may potentially affect disease progression in BMD, including mechanical stress caused by strenuous muscle exercise,^55^ previous findings combined with our current findings suggest that altered expression of collagens as a result of the second-hit *PLOD3* variant may be at least in part responsible for the underlying molecular basis of the advanced HF observed in the proband.

### Study limitations

Because iPSC-CMs differentiated under in vitro conditions do not fully resemble in vivo heart tissue,^56^ further attempts to improve the maturity of iPSC-CMs will be required to recapitulate the pathogenesis of HF observed in the proband. Although our functional analysis using SOTRs demonstrated that decreased LH3 expression combined with Δ45-48 truncated dystrophin exacerbated myocardial injury, further experiments using an in vivo model will be required to reveal the molecular basis underlying advanced HF observed in the proband. The heterozygous *PLOD3* Y630X variant identified in the proband is an extremely rare variant with an allele frequency of 0.0000199 identified in 5 of 251,218 alleles in the Genome Aggregation Database (gnomAD database),^57^ thereby suggesting that the pathologic effect of the combined genetic variants shown in this study does not necessarily have global implications for other BMD carriers with advanced HF.

BMD carriers are not eligible for the latest therapeutics because developing gene therapies target the excision or skipping of the mutated exon to restore the in-frame expression of dystrophin protein.^3^ Our findings using isogenic iPSC-CMs shed new light on the underlying molecular basis of advanced HF and may provide a potential therapeutic target for female BMD carriers.

Perspectives **COMPETENCY IN MEDICAL KNOWLEDGE:** Female BMD carriers rarely develop early onset advanced HF. Functional analysis using isogenic iPSC-CMs recapitulated the reduced contractility and stiffness caused by combined variants in *DMD* and *PLOD3* and provided insight into the molecular basis underlying advanced HF.

**TRANSLATIONAL OUTLOOK:** BMD patients are not currently eligible for the latest therapeutics because the developing gene therapies for DMD aim to restore in-frame expression of the dystrophin protein. Our current findings using isogenic iPSC-CMs provide a potential therapeutic target other than the *DMD* variant.

## Funding Support and Author Disclosures

This work was supported by JSPS KAKENHI grant numbers 19K08489, 19K12801, 20K21602, 21H02915, and 22K19526, grants-in-aid from the Japanese Ministry of Health, Labor, and Welfare, the Japan agency for medical research and development (21bm0804008h0005, 22bm0804035h0001). This work was also supported by the Cell Science Research Foundation and the Grant for Basic Research of the Japanese Circulation Society (2018), SENSHIN Medical Research Foundation, Daiichi Sankyo, Mitsubishi Tanabe Pharma, and the Center of Medical Innovation and Translational Research and the Center for Medical Research and Education of the Graduate School of Medicine, Osaka University. The Department of Medical Therapeutics for Heart Failure was an endowment department supported by Actelion Pharmaceuticals Japan (2015-2020); the Department of Medical Therapeutics for Heart Failure is currently a Joint Research Department with TOA EIYO Pharmaceutical Company. The authors have reported that they have no relationships relevant to the contents of this paper to disclose.

## References

[1] Blake D.J., Weir A., Newey S.E., Davies K.E. (2002). Function and genetics of dystrophin and dystrophin-related proteins in muscle. Physiol Rev.

[2] Mah J.K., Korngut L., Dykeman J., Day L., Pringsheim T., Jette N. (2014). A systematic review and meta-analysis on the epidemiology of Duchenne and Becker muscular dystrophy. Neuromuscul Disord.

[3] Duan D., Goemans N., Takeda S., Mercuri E., Aartsma-Rus A. (2021). Duchenne muscular dystrophy. Nat Rev Dis Primers.

[4] Broomfield J., Hill M., Guglieri M., Crowther M., Abrams K. (2021). Life Expectancy in Duchenne muscular dystrophy: reproduced individual patient data meta-analysis. Neurology.

[5] Ho R., Nguyen M.L., Mather P. (2016). Cardiomyopathy in becker muscular dystrophy: overview. World J Cardiol.

[6] Yilmaz A., Gdynia H.J., Baccouche H. (2008). Cardiac involvement in patients with Becker muscular dystrophy: new diagnostic and pathophysiological insights by a CMR approach. J Cardiovasc Magn Reson.

[7] Hoogerwaard E.M., van der Wouw P.A., Wilde A.A. (1999). Cardiac involvement in carriers of Duchenne and Becker muscular dystrophy. Neuromuscul Disord.

[8] Grain L., Cortina-Borja M., Forfar C., Hilton-Jones D., Hopkin J., Burch M. (2001). Cardiac abnormalities and skeletal muscle weakness in carriers of Duchenne and Becker muscular dystrophies and controls. Neuromuscul Disord.

[9] Schade van Westrum S.M., Hoogerwaard E.M., Dekker L. (2011). Cardiac abnormalities in a follow-up study on carriers of Duchenne and Becker muscular dystrophy. Neurology.

[10] McCaffrey T., Guglieri M., Murphy A.P., Bushby K., Johnson A., Bourke J.P. (2017). Cardiac involvement in female carriers of duchenne or becker muscular dystrophy. Muscle Nerve.

[11] Holloway S.M., Wilcox D.E., Wilcox A. (2008). Life expectancy and death from cardiomyopathy amongst carriers of Duchenne and Becker muscular dystrophy in Scotland. Heart.

[12] Pioner J.M., Fornaro A., Coppini R. (2020). Advances in stem cell modeling of dystrophin-associated disease: implications for the wider world of dilated cardiomyopathy. Front Physiol.

[13] Gowran A., Spaltro G., Casalnuovo F. (2018). Generation of induced pluripotent stem cells from a Becker muscular dystrophy patient carrying a deletion of exons 45-55 of the dystrophin gene (CCMi002BMD-A-9 45-55). Stem Cell Res.

[14] Rovina D., Castiglioni E., Niro F. (2020). Generation of the Becker muscular dystrophy patient derived induced pluripotent stem cell line carrying the DMD splicing mutation c.1705-8 T>C. Stem Cell Res.

[15] Wutz A. (2011). Gene silencing in X-chromosome inactivation: advances in understanding facultative heterochromatin formation. Nat Rev Genet.

[16] Komoriyama H., Fukushima A., Takahashi Y. (2019). Rapidly progressive heart failure in a female carrier of Becker muscular dystrophy with no skeletal muscle symptoms. Intern Med.

[17] Higo S., Hikoso S., Miyagawa S., Sakata Y. (2021). Genome editing in human induced pluripotent stem cells (hiPSCs). Methods Mol Biol.

[18] Inoue H., Nakamura S., Higo S. (2022). Modeling reduced contractility and impaired desmosome assembly due to plakophilin-2 deficiency using isogenic iPS cell-derived cardiomyocytes. Stem Cell Reports.

[19] Li J., Zhang L., Yu L. (2020). Circulating re-entrant waves promote maturation of hiPSC-derived cardiomyocytes in self-organized tissue ring. Commun Biol.

[20] Shiba M., Higo S., Kondo T. (2021). Phenotypic recapitulation and correction of desmoglein-2-deficient cardiomyopathy using human-induced pluripotent stem cell-derived cardiomyocytes. Hum Mol Genet.

[21] Viggiano E., Picillo E., Cirillo A., Politano L. (2013). Comparison of X-chromosome inactivation in Duchenne muscle/myocardium-manifesting carriers, non-manifesting carriers and related daughters. Clin Genet.

[22] Viggiano E., Picillo E., Ergoli M., Cirillo A., Del Gaudio S., Politano L. (2017). Skewed X-chromosome inactivation plays a crucial role in the onset of symptoms in carriers of Becker muscular dystrophy. J Gene Med.

[23] Lee Y.T., Sung K., Shin J.O., Jeon E.S. (2006). Images in cardiovascular medicine. Disruption of dystrophin in acute fulminant coxsackieviral B4 infection. Circulation.

[24] Wasala N.B., Lai Y., Shin J.H., Zhao J., Yue Y., Duan D. (2016). Genomic removal of a therapeutic mini-dystrophin gene from adult mice elicits a Duchenne muscular dystrophy-like phenotype. Hum Mol Genet.

[25] Tanihata J., Nagata T., Ito N. (2018). Truncated dystrophin ameliorates the dystrophic phenotype of mdx mice by reducing sarcolipin-mediated SERCA inhibition. Biochem Biophys Res Commun.

[26] Bittel D.C., Theodoro M.F., Kibiryeva N., Fischer W., Talebizadeh Z., Butler M.G. (2008). Comparison of X-chromosome inactivation patterns in multiple tissues from human females. J Med Genet.

[27] Suwa Y., Higo S., Nakamoto K. (2019). Old-age onset progressive cardiac contractile dysfunction in a patient with polycystic kidney disease harboring a PKD1 frameshift mutation. Int Heart J.

[28] Qi Y., Xu R. (2018). Roles of PLODs in collagen synthesis and cancer progression. Front Cell Dev Biol.

[29] Ewans L.J., Colley A., Gaston-Massuet C. (2019). Pathogenic variants in PLOD3 result in a Stickler syndrome-like connective tissue disorder with vascular complications. J Med Genet.

[30] Scietti L., Chiapparino A., De Giorgi F. (2018). Molecular architecture of the multifunctional collagen lysyl hydroxylase and glycosyltransferase LH3. Nat Commun.

[31] Salo A.M., Cox H., Farndon P. (2008). A connective tissue disorder caused by mutations of the lysyl hydroxylase 3 gene. Am J Hum Genet.

[32] Vahidnezhad H., Youssefian L., Saeidian A.H. (2019). Mutations in PLOD3, encoding lysyl hydroxylase 3, cause a complex connective tissue disorder including recessive dystrophic epidermolysis bullosa-like blistering phenotype with abnormal anchoring fibrils and type VII collagen deficiency. Matrix Biol.

[33] Tchieu J., Kuoy E., Chin M.H. (2010). Female human iPSCs retain an inactive X chromosome. Cell Stem Cell.

[34] Burridge P.W., Matsa E., Shukla P. (2014). Chemically defined generation of human cardiomyocytes. Nat Methods.

[35] Mekhoubad S., Bock C., de Boer A.S., Kiskinis E., Meissner A., Eggan K. (2012). Erosion of dosage compensation impacts human iPSC disease modeling. Cell Stem Cell.

[36] Kuramoto Y., Naito A.T., Tojo H. (2018). Generation of Fabry cardiomyopathy model for drug screening using induced pluripotent stem cell-derived cardiomyocytes from a female Fabry patient. J Mol Cell Cardiol.

[37] Vallot C., Ouimette J.F., Makhlouf M. (2015). Erosion of X chromosome inactivation in human pluripotent cells initiates with XACT coating and depends on a specific heterochromatin landscape. Cell Stem Cell.

[38] Ortiz-Vitali J.L., Darabi R. (2019). iPSCs as a platform for disease modeling, drug screening, and personalized therapy in muscular dystrophies. Cells.

[39] Tulloch N.L., Muskheli V., Razumova M.V. (2011). Growth of engineered human myocardium with mechanical loading and vascular coculture. Circ Res.

[40] Yang K.C., Breitbart A., De Lange W.J. (2018). Novel adult-onset systolic cardiomyopathy due to MYH7 E848G mutation in patient-derived induced pluripotent stem cells. J Am Coll Cardiol Basic Trans Science.

[41] Wang C., Risteli M., Heikkinen J., Hussa A.K., Uitto L., Myllyla R. (2002). Identification of amino acids important for the catalytic activity of the collagen glucosyltransferase associated with the multifunctional lysyl hydroxylase 3 (LH3). J Biol Chem.

[42] Matsuura K., Seta H., Haraguchi Y. (2016). TRPV-1-mediated elimination of residual iPS cells in bioengineered cardiac cell sheet tissues. Sci Rep.

[43] Heikkinen J., Risteli M., Wang C. (2000). Lysyl hydroxylase 3 is a multifunctional protein possessing collagen glucosyltransferase activity. J Biol Chem.

[44] Piek A., de Boer R.A., Sillje H.H. (2016). The fibrosis-cell death axis in heart failure. Heart Fail Rev.

[45] Kato S., Spinale F.G., Tanaka R., Johnson W., Cooper G.T., Zile M.R. (1995). Inhibition of collagen cross-linking: effects on fibrillar collagen and ventricular diastolic function. Am J Physiol.

[46] Deacon D.C., Happe C.L., Chen C. (2019). Combinatorial interactions of genetic variants in human cardiomyopathy. Nat Biomed Eng.

[47] Morimoto Y., Chonabayashi K., Kawabata H. (2022). Azacitidine is a potential therapeutic drug for pyridoxine-refractory female X-linked sideroblastic anemia. Blood Adv.

[48] Barnabei M.S., Metzger J.M. (2012). Ex vivo stretch reveals altered mechanical properties of isolated dystrophin-deficient hearts. PLoS One.

[49] Ruotsalainen H., Sipila L., Vapola M. (2006). Glycosylation catalyzed by lysyl hydroxylase 3 is essential for basement membranes. J Cell Sci.

[50] Sipila L., Ruotsalainen H., Sormunen R. (2007). Secretion and assembly of type IV and VI collagens depend on glycosylation of hydroxylysines. J Biol Chem.

[51] Valera I.C., Wacker A.L., Hwang H.S. (2021). Essential roles of the dystrophin-glycoprotein complex in different cardiac pathologies. Adv Med Sci.

[52] Marijianowski M.M.H., Teeling P., Mann J., Becker A.E. (1995). Dilated cardiomyopathy is associated with an increase in the type I/type III collagen ratio: a quantitative assessment. J Am Coll Cardiol.

[53] Uchinaka A., Yoshida M., Tanaka K. (2018). Overexpression of collagen type III in injured myocardium prevents cardiac systolic dysfunction by changing the balance of collagen distribution. J Thorac Cardiovasc Surg.

[54] Weber K.T. (1989). Cardiac interstitium in health and disease: the fibrillar collagen network. J Am Coll Cardiol.

[55] Melacini P., Fanin M., Danieli G.A. (1996). Myocardial involvement is very frequent among patients affected with subclinical Becker's muscular dystrophy. Circulation.

[56] Sayed N., Liu C., Wu J.C. (2016). Translation of human-induced pluripotent stem cells: from clinical trial in a dish to precision medicine. J Am Coll Cardiol.

[57] Karczewski K.J., Francioli L.C., Tiao G. (2020). The mutational constraint spectrum quantified from variation in 141,456 humans. Nature.

