# Supplementary material for: Modeling Reduced Contractility and Stiffness Using iPSC-Derived Cardiomyocytes Generated From Female Becker Muscular Dystrophy Carrier
Source: JACC Basic Transl Sci. 2023 Feb 8;8(6):599–613. doi: 10.1016/j.jacbts.2022.11.007 (PMC10322885; doi:10.1016/j.jacbts.2022.11.007)
Supplement: Supplemental Appendix [file mmc1.pdf]

SUPPLEMENTAL APPENDIX

Supplemental Methods, Figures and Table **Page**

Supplemental Methods 2

Supplemental Figure 1 11

Supplemental Figure 2 12

Supplemental Figure 3 14

Supplemental Figure 4 15

Supplemental Figure 5 17

Supplemental Table 19

Supplemental References 21

Supplemental Methods

Generation of human iPSC clones. Patient-derived iPSC clones were established from peripheral blood mononuclear cells (PBMCs) taken from the patient as previously described.[1, 2] Briefly, PBMCs were isolated from peripheral whole blood using Ficoll-Plaque (GE). Sendai virus vectors, OCT3/4, SOX2, KLF4 and c-MYC (CytoTune-iPS 2.0 Sendai Reprogramming Kit, Life Technologies), were infected for reprograming. Twenty-four hours after infection, PBMCs were seeded on laminin-coated plate (iMatrix-511, MATRIXOME, Osaka, Japan). iPS cells were maintained on laminin-coated plate with medium (StemFit AK02, AJINOMOTO). To identify whether iPSC expresses the paternal (Δ45–48 dystrophin) or maternal (WT) X chromosome, we evaluated *DMD* transcripts by droplet digital PCR (ddPCR) after cardiomyocyte differentiation. Control iPSC clone was generated from a female healthy subject without heart disease.

Transfection of plasmids into human iPSCs and selection of the targeted clones. Plasmid constructs for genome editing were transfected into iPSCs as described previously[2, 3] with some modifications. A suspension of iPSCs (1×10^5 cells / 20μl) was mixed with 2 μg pX459 vector encoding gRNA against *PLOD3* sequence and 2 μg repair template DNA (pCR bluntⅡ vector, Thermo) to introduce non-homologous end joining (NHEJ) or homology-directed repair (HDR) in *PLOD3*. 40μl of the mixture was transferred to NEPA 21 electroporator (Nepagene) and electroporated (poring pulse: pulse voltage, 125 V; pulse width, 5 ms; pulse number, 2). Twenty-four hours after electroporation,

puromycin (0.2 μg/ml) was added. Twenty-four hours after puromycin addition, the culture medium was replaced with puromycin-free medium. Seven days later, iPSCs were passaged into 6-well plates at a density of 500 cells/well for clonal colony formation. Picked up colonies were split into two 96-well plates for genotyping and cell expansion. The target genomic region was amplified by PCR, and then evaluated by direct sequencing or sequence analysis after cloning.

Chemically defined differentiation of human iPSC-CMs. The human iPSCs were differentiated into human iPSC-CMs using a chemically defined protocol.[4] The culture medium was exchanged for a differentiation medium that contained RPMI 1640 medium (thermoFisher Scientific, USA), recombinant human albumin (Sigma-Aldrich) and L-ascorbic acid 2-phosphate (Sigma-Aldrich). iPSCs were treated with CHIR99021 (LC Laboratories, USA) (day 0 to 2), Wnt-C59 (Selleck Chemicals, USA) and XAV-939 (Cayman, USA) (day 2 to 4). When replating, iPSC-CMs were dissociated with 0.25% Trypsin-EDTA (Gibco, USA) and reseeded on Gelatin-coated plates.

Generation of self-organized tissue rings (SOTRs). Three-dimensional SOTRs were generated as described previously.[1, 5] Before seeding into culture plates, single CMs were filtered using a 40-μm cell strainer (BD Falcon; Becton Dickenson, Franklin Lakes, NJ, USA) and resuspended at a density of 2×10^6 cells/mL in serum-supplemented cardiac differentiation culture medium containing 40% high glucose Dulbecco's Modified Eagle Medium (DMEM; Sigma-Aldrich), 40% Iscove's Modified

Dulbecco's Medium (IMDM; Sigma-Aldrich), 20% fetal bovine serum (FBS; Gibco, USA), 1% minimum essential medium non-essential amino acid solution (Sigma-Aldrich), 0.1% penicillin-streptomycin (Gibco, USA), and 0.5% L-glutamine (Sigma-Aldrich). Cells were plated into each PDMS culture well with a 3-mm pillar (7×10^5 cells/well). Within 7 days after plating, the CMs settled in the wells, aggregated, and congregated around the central pillar to form densely packed tissue rings. The medium was changed to a serum-free medium starting on day 2 and changed with fresh medium every 4 days.

Force testing using MicroTester G2. The active force and stiffness of SOTRs were measured using a micron-scale mechanical testing system (MicroTester G2; CellScale Biomaterials Testing, Waterloo, ON, Canada) as previously described.[1, 5] The SOTRs were removed from the pillars, hanged on the MicroTester G2 hooks, and immersed in culture medium at 37 °C. A cantilever beam (0.30 mm diameter) was pressed onto the SOTR from the opposite side of the hook. The length was increased by 2 mm over 30 s and then held for 30 s as 1 cycle. The cycles were continued until the hanged SOTR broke. The active forces were analyzed as the maximum amplitude of the recorded twitch force and normalized to the cross-sectional area (CSA). To analyze the stiffness of the SOTRs, the diastolic forces per CSA were recorded 15 s after the beam was moved and the value adopted as the passive force. Passive stiffness was defined by the slope of the diastolic forces per CSA versus the length curve according to the Young's Modulus.[6, 7] Force and stiffness were calculated by our custom-made

MATLAB scripts (R2019a; Math Works, Natick, MA, USA).

Generation of human iPSC-CFs. The human iPSCs were differentiated into human iPSC-cardiac fibroblasts according to the previous report.[8, 9] Briefly, human iPSCs was seeded on laminin-coated 12-well plates at a density of 6 × 10^6 cells/well in StemFit medium. iPSCs were cultured in medium for 4 days until they reached 100% confluency. At day 0 the medium was changed to RPMI + B27 without insulin and 6 μM CHIR99021 was added and cells were incubated for 24 hours (day 1). The next day, the medium was changed to RPMI + B27 without insulin and incubated for 48 hours (day 2–3). At day 3, the medium was changed to CFBM medium with 75 ng/ml bFGF (R&D Systems, Minneapolis, MN, USA). Cells were cultured with CFBM with bFGF every other day until day 20 for analysis. At day 20, iPSC-CFs were dissociated using 0.05% Trypsin-EDTA (Gibco, USA) and reseeded on non-coating plastic plates in FibroGRO medium (Millipore EMD, SCMF001) + 2% FBS (Sigma-Aldrich). Each analysis was performed using iPSC-CFs after day 30 of differentiation.

Immunohistochemical staining. The myocardial samples obtained at the implantation of LVAD were fixed in 10% neutral buffered formalin for 24 hours and embedded in paraffin. Serial cross-sections (4 μm thickness) were stained for hematoxylin and eosin, and Masson's trichrome, or for immunohistochemistry (IHC). The following primary antibodies were used in IHC: mouse anti-human dystrophin monoclonal antibody (NCL-DYSA, 1:20, Leica Biosystems Newcastle Ltd, UK), mouse

anti-human dystrophin (NCL-DYSB, 1:20, Leica Biosystems Newcastle Ltd, UK) and MANEX 45A (1:20, Development Studies Hybridoma Bank, Iowa) were used to detect the rod domain, amino acids 321 to 494 and exon 45 of the human dystrophin molecule, respectively.

Amplicon sequence analysis. The genomic DNA was extracted from the peripheral blood of the patient using QIAAmp DNA mini kit (QIAGEN). We prepared genomic DNA library using Ion AmpliSeq Library Kit and Ion Ampliseq Cardiovascular Research Panel (10,430 PCR amplicons covering 404 genes known to harbor variants affecting cardiovascular function).[10] Variants with low quality score less than 30 or with low read depth less than 30 were excluded. Among the identified exonic variants, synonymous variants without amino acid changes were excluded. Variants were classified to be benign when present in Human Genetic Variation Database (HGVD)[11] or ESP 6500[12] database with an allele frequency of more than 0.01. After filtering, two heterozygous nonsynonymous variants in *TTN* and one heterozygous stop-gain variant in *PLOD3* were identified only in the proband but not in her non-manifesting sister. The stop-gain variant in *PLOD3* (c.T1890G, p.Y630X) with scaled CADD (CADD_Phred) score of 41 was considered to be a pathogenic variant.

X chromosome inactivation assays. The XCI assays were performed as reported previously.[13, 14] Genomic DNA was extracted from peripheral blood cells collected from the proband, her sister, and brother using a QIAamp DNA Mini Kit (QIAGEN). An aliquot of the genomic DNA (50 ng) was

digested with 1 unit of HpaII restriction endonuclease at 37 °C overnight. An equal volume of DNA was incubated overnight without HpaII and used as a control. After phenol and chloroform extraction, 5 ng of digested or non-digested DNA samples were used as template for PCR amplification of a region of exon 1 of the androgen receptor (AR). The PCR primers are listed in the Supplementary Table. PCR cycling conditions were 94°C, 2 min, followed by 33 cycles of 98 °C 10 s, 71 °C 30 s, 68 °C 30 s. The PCR fragments were purified and analyzed using an MCE-202 MultiNa Microchip Electrophoresis System (Shimadzu Biotech, Kyoto, Japan) with a DNA-500 Reagent Kit.[15] The degree of XCI in the digested DNA was calculated as the peak area of (WT digested/WT non-digested) or (Δ45–48 digested/Δ45–48 non-digested)/(WT digested/WT non-digested) + (Δ45–48 digested/Δ45–48 non-digested) × 100. The degree of activated WT or Δ45–48 *DMD* in the X chromosome was estimated as (100 – the degree of XCI in the digested DNA).

Droplet digital PCR and quantitative real-time PCR. Droplet digital PCR (ddPCR) was performed as described previously[2] using the QX200 ddPCR system (Bio-Rad). To specifically detect transcripts from the WT allele or Δ45–48 allele in *DMD*, HEX- or FAM-labeled probes were designed (Bio-Rad). The probes for WT allele and Δ45–48 allele were designed to span Ex44 to 45 and Ex44 to 49, respectively. Probe sequences are listed in Supplementary Table. After PCR, each generated droplets were individually detected for fluorescence and analyzed by a QX200 droplet reader (Bio-Rad).

Generation and purification of adeno-associated viruses (AAV). The full length human *B4GALT1* coding sequence was cloned from human cDNA using PCR primers and the *B4GALT1-EGFP* sequence was then subcloned into a pAAV cytomegalovirus (CMV) vector. HEK293T cells were transfected with pAAV vector, pHelper vector, and a pRC2-mi342 vector (AAVpro Helper Free System, TaKaRa) using calcium phosphate transfection (CalPhos Mammalian Transfection Kit, TaKaRa) according to the manufacture's protocol. Seventy-two hours after transfection, 1/80 volume of 0.5 mol/L EDTA (pH 8.0) was added to the transfected cells. The HEK293T cells were detached and pelleted by low-speed centrifugation at 2000 x *g* for 10 min. The cell pellets were lysed with AAV Extraction Solution A, centrifuged at 9000 x *g* for 10 min, and the supernatant collected. AAV Extraction Solution B was then added to the supernatant and stored at −80°C. The collected AAV was purified using an AAVpro Purification Kit (TaKaRa) and the viral titers calculated using an AAV Titration Kit (TaKaRa).

Western blotting. Cells were washed with cold PBS and directly lysed with CHAPS Lysis buffer (1% CHAPS, 25 mmol/L Tris-HCl (pH7.4), 137 mmol/L NaCl, 2.68 mmol/L KCl, 5 mmol/L EDTA). Dystrophin expression was evaluated in iPSC-CMs 30–40 days after differentiation according to the previous report.[16, 17] The protein concentration was determined by BCA Protein Assay Kit (Thermo). Lysate samples were mixed with 4 × Laemmli sample buffer (Bio-Rad) with mercaptoethanol (2.5%). Proteins were separated by SDS-PAGE and transferred to PVDF membrane. After blocking with 1%

Bovine Serum Albumin or 3% nonfat milk for 1 h, the transferred membrane was incubated with primary antibody overnight at 4°C and with secondary antibody for 45 min at room temperature. The chemiluminescent signals were detected using ECL or ECL prime reagent (GE). The protein expression level was quantified using ImageQuant TL (GE). The expression level of each protein was normalized by that of GAPDH.

Immunofluorescence. Cardiomyocytes were fixed in 4% PFA for 15 min at room temperature. Cells were washed in PBS and permeabilized with 0.5% Triton-X 100 for 20 min. 1% BSA in PBS was used to block nonspecific antibody binding for 1h at room temperature. After blocking, cells were incubated in primary antibodies diluted with 1% BSA in PBS overnight at 4°C. After incubation with primary antibodies, an Alexa 488-, 568- or 647- labeled secondary antibodies (Invitrogen) were diluted in blocking solution and reacted for more than 30 min.

Reagents and antibodies. OCT-3/4 (C-10) (Santa Cruz Biotechnology, Dallas, Texas, USA, Cat# sc-5279, RRID: AB_628051), TRA-1-60 (Merck Millipore, Burlington, Massachusetts, USA, Cat# MAB4360, RRID: AB_2119183), SSEA-4 (Merck Millipore, Cat# MAB4304, RRID: AB_177629), NANOG (Abcam, Cambridge, MA, USA, Cat# ab80892, RRID: AB_2150114), Dystrophin (Abcam, Cat#ab15277, RRID:AB_301813), Dystrophin (Novocastra, Newcastle, UK, Cat# NCL-DYSA, RRID:AB_563692), Dystrophin (Novocastra, Newcastle, UK, Cat# NCL-DYSB, RRID:AB_563691),

MANEX45A (Development Studies Hybridoma Bank, Cat#8F10, RRID:AB_2618177), PLOD3 (Proteintech, Rosemont, IL, USA, Cat#11027-1-AP, RRID:AB_2165781), Collagen Type Ⅰ (Proteintech, Rosemont, IL, USA, Cat#14695-1-AP, RRID:AB_2082037), Collagen Type Ⅲ (Proteintech, Rosemont, IL, USA, Cat#22734-1-AP, RRID:AB_2879158), Troponin T-C (CT3) (Santa Cruz Biotechnology, Cat# sc-20025, RRID: AB_628403), GAPDH (Santa Cruz Biotechnology, Cat# sc-47724, RRID: AB_627678), Sarcomeric Alpha Actinin (EA-53) (Abcam, Cat# ab9465, RRID: AB_307264), Hoechst 33342 (DOJINDO Molecular Technologies, Kumamoto, Japan, Cat# H342), Puromycin dihydrochloride (SIGMA, Cat# P9620-10ML).

RNA extraction and PCR. Total RNA was extracted using RNeasy mini kit (QIAGEN) from LV tissue, iPSCs and iPSC-CMs. A high capacity RNA-to cDNA RT kit (Thermo) was used to generate cDNA from total RNA. These cDNA were used in PCR, quantitative real-time PCR, and droplet digital PCR analysis. Primer sequences are listed in Supplementary Table. To amplify the targeted sequence spanning Δ45–48 in *DMD*, PCR was performed as follows: 94°C for 2 min, followed by 33 cycles of 98°C for 10 s, 60℃ for 30 s (KOD Fx Neo, TOYOBO). PCR products were purified using Gel Extraction kit (QIAGEN), and the deletion site was evaluated by direct sequencing. Quantitative real-time PCR was performed using SYBR green method (THUNDERBIRD SYBR qPCR mix, TOYOBO). All samples were processed in duplicate. mRNA expression levels of each transcript were normalized by *GAPDH*. PCR primers used for quantitative PCR are listed in Supplementary Table.

Supplemental Figure 1. Immunohistochemical Analysis of a Patient with No History of Cardiac Disease and Echocardiographic Findings of the Proband's Sister (III-2) at 36 Years of Age.

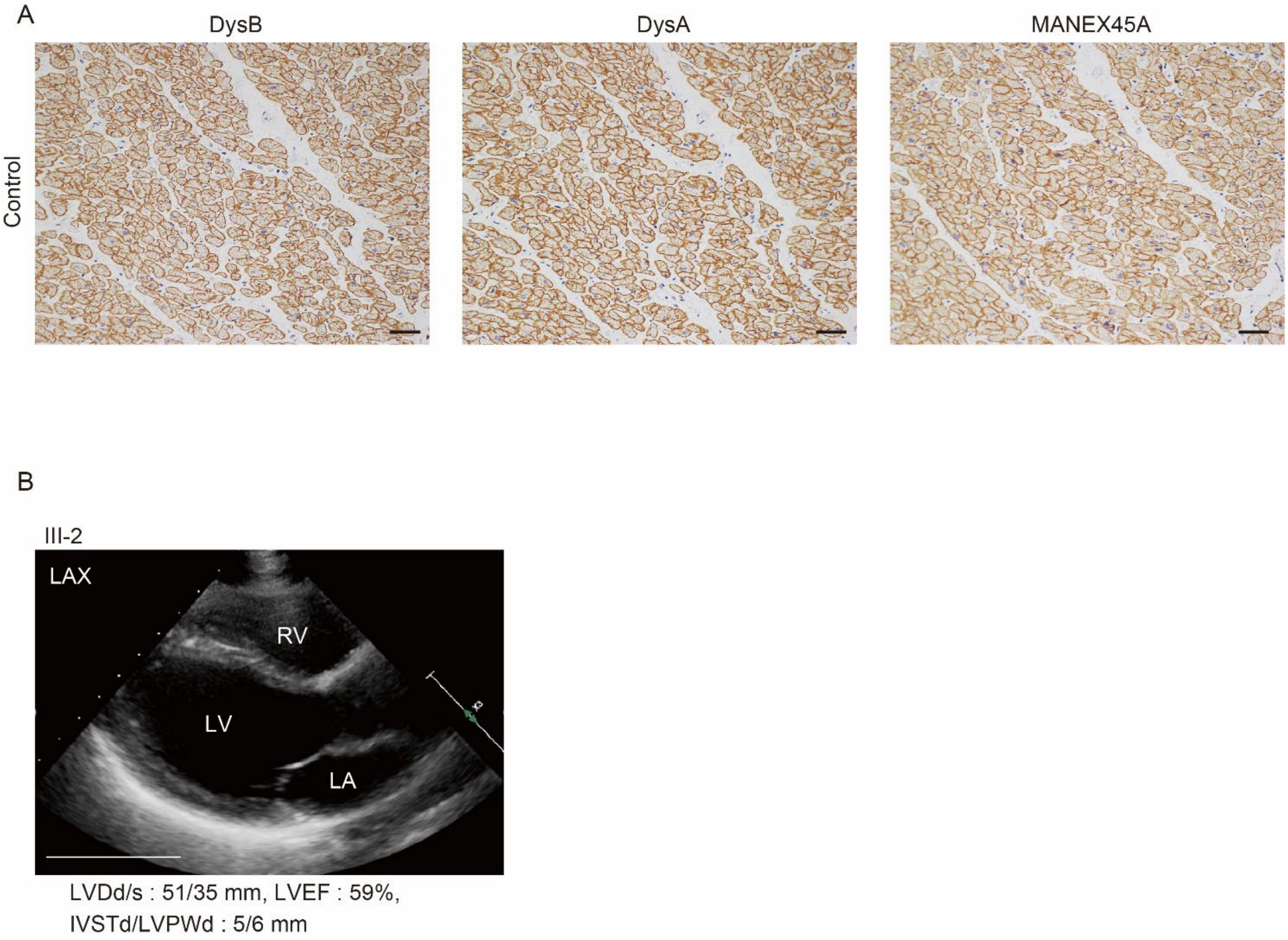

(A) Immunohistochemical analysis of the left ventricular myocardium obtained from a patient with no history of cardiac disease using the indicated antibodies. Scale bars; 50 µm. **(B)** LAX, left parasternal long axis view; LV, left ventricle; LA, left atrium; RV right ventricle; LVDd, left ventricular diastolic diameter; LVDs, left ventricular systolic diameter; LVEF, left ventricular ejection fraction; IVSTd, interventricular septal thickness at end-diastole; LVPWd, left ventricular posterior wall thickness at end-diastole. Scale bar: 50 mm.

Supplemental Figure 2. Expression of Pluripotent Markers, Karyotype Analysis, and Erosion Analysis of WT-*DMD* and Δ45–48-*DMD* iPSCs.

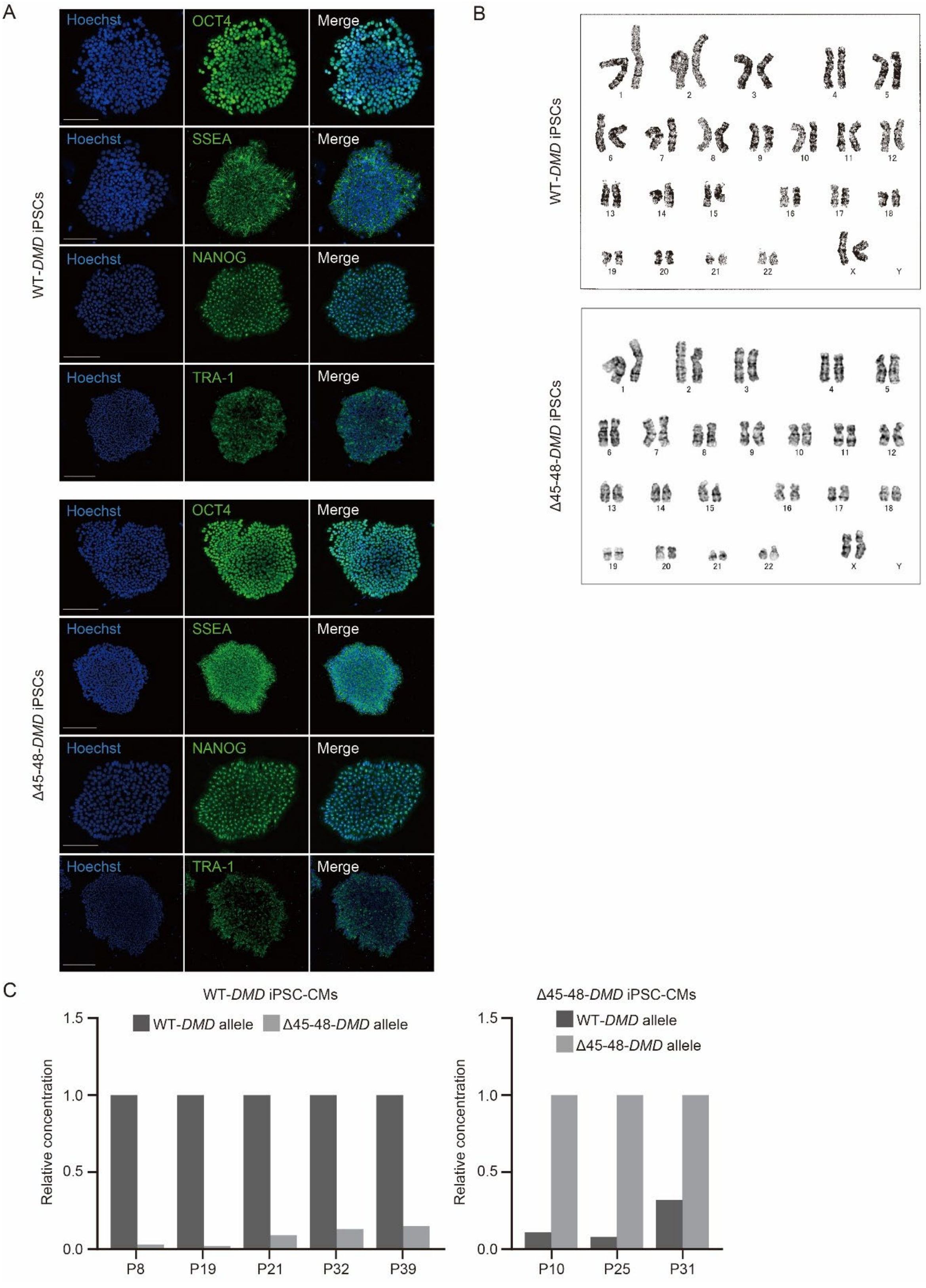

(A) Immunofluorescence staining of pluripotent markers in WT-*DMD* and Δ45–48-*DMD* iPSCs using the indicated antibodies. Nuclei were detected using Hoechst staining. Scale bars; 100 μm. **(B)** Karyotype analysis of patient-derived WT-*DMD* and Δ45–48-*DMD* iPSCs. **(C)** Results of ddPCR analysis using cDNA samples obtained from WT-*DMD* and Δ45–48-*DMD* iPSC-CMs differentiate from iPSCs after the indicated number of passages. The concentration of each transcript (copies/μL) was normalized to that of the dominant transcript.

Supplemental Figure 3. Schematic of Genome Editing of *PLOD3* in Δ45–48-*DMD* and Δ45–48-*DMD*-HDR iPSCs and a Plot of the Diastolic Force/CSA.

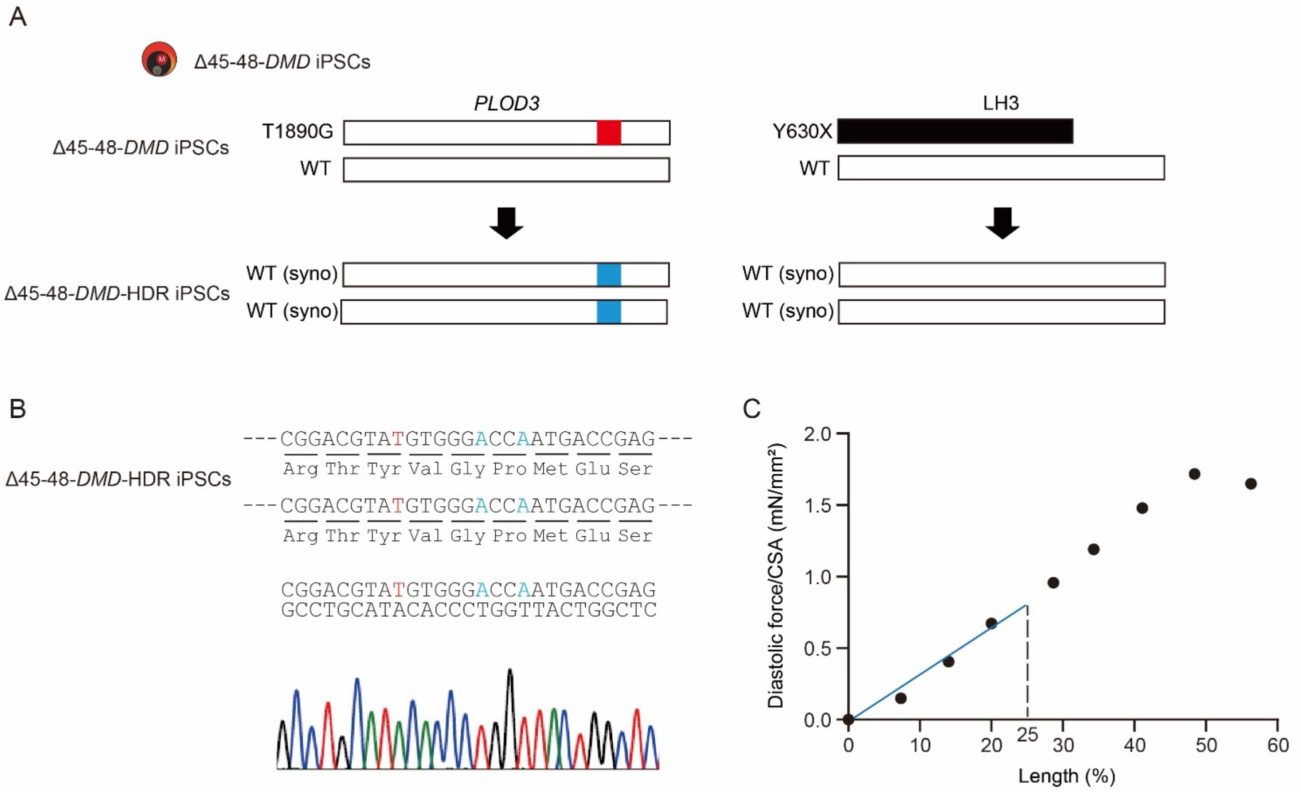

(A) Schematic of the genomic sequence of *PLOD3* (left) and the translated amino-acid sequence of LH3 (right) in Δ45–48-*DMD* and Δ45–48-*DMD*-HDR iPSCs. Homozygous corrected Δ45–48-*DMD*-HDR iPSCs carry sequence with synonymous variants (syno). **(B)** Result of direct sequence proximal to T1890 of *PLOD3* in Δ45–48-*DMD*-HDR iPSCs. The corrected T1890G variant is highlighted in red and synonymous variants are highlighted in blue. **(C)** A representative plot of the diastolic force per CSA. The diastolic force was normalized to CSA and plotted. Passive stiffness was calculated as the slope (blue line) determined according to the diastolic force to length ratio at the first 25% of the initial length.

Supplemental Figure 4. Schematic of the Genome Editing and Quantitative PCR Analysis of *PLOD3* in WT-*DMD* and WT-*DMD*-NHEJ iPSCs.

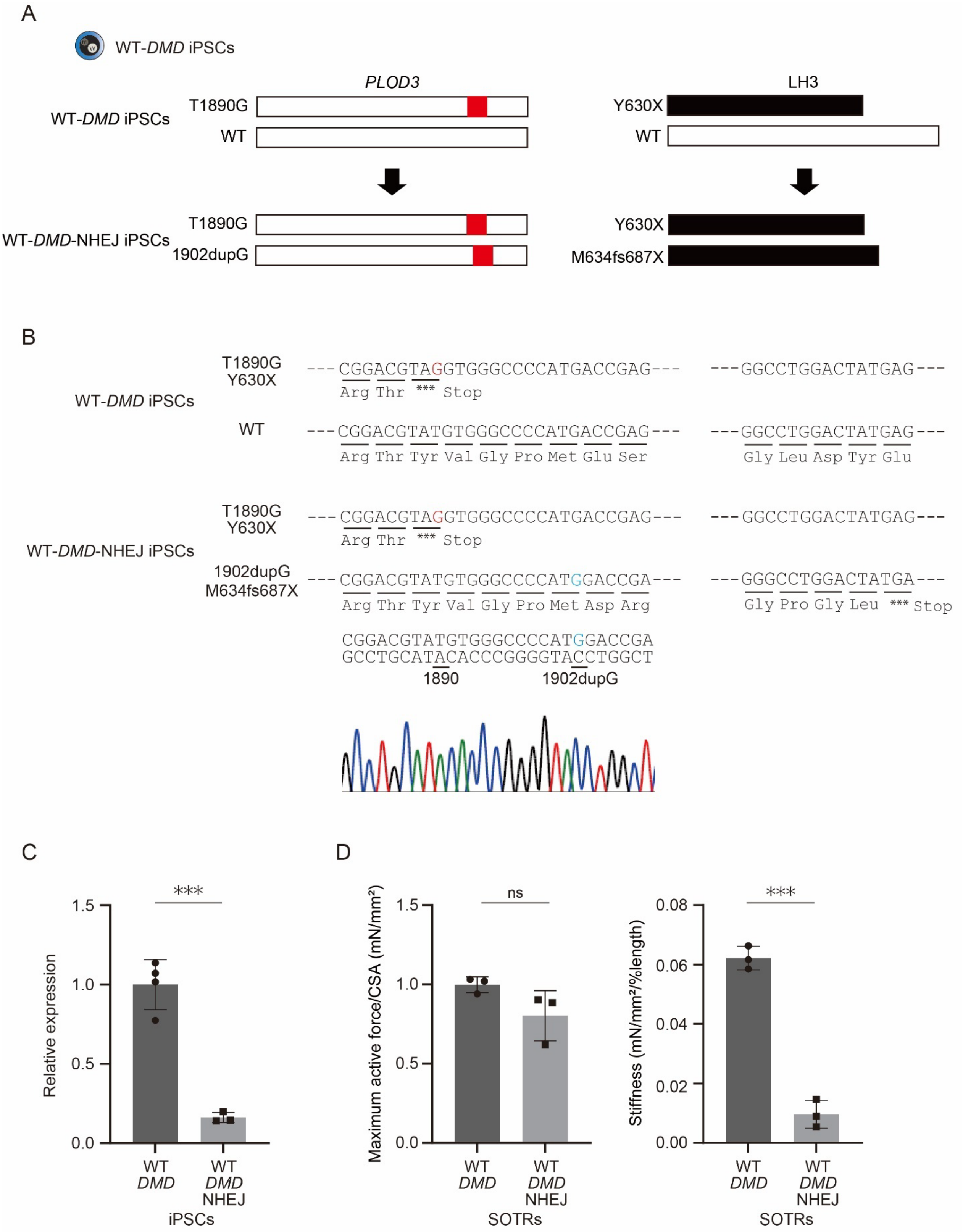

(A) Schematic of the genomic sequence of *PLOD3* (left) and the translated amino-acid sequence of LH3 (right) in WT-*DMD* iPSCs and WT-*DMD*-NHEJ iPSCs. **(B)** Result of Sanger sequence proximal to T1890 of *PLOD3* in the edited allele of WT-*DMD*-NHEJ iPSCs. An insertion variant (1902dupG) led to a frameshift variant and protein termination at amino-acid residue 687. The T1890G variant is highlighted in red. The 1902dupG insertion is highlighted in blue. **(C)** The relative expression levels of *PLOD3* normalized by *GAPDH* were measured using quantitative PCR in WT-*DMD* iPSCs (n = 4) and WT-*DMD*-NHEJ iPSCs (n = 3). Relative expression levels normalized to the levels of WT-*DMD* iPSCs are shown. ***$P < 0.001$. **(D)** Maximum active force corrected by CSA and stiffness were compared between WT-*DMD* (n = 3) and WT-*DMD*-NHEJ SOTRs (n = 3). ***$P<0.001$, ns = not significant.

Supplemental Figure 5. Generation of Cardiac Fibroblasts From iPSCs (iPSC-CFs) and Quantitative PCR Analysis of CF markers in WT-*DMD* and WT-*DMD*-NHEJ iPSC-CFs.

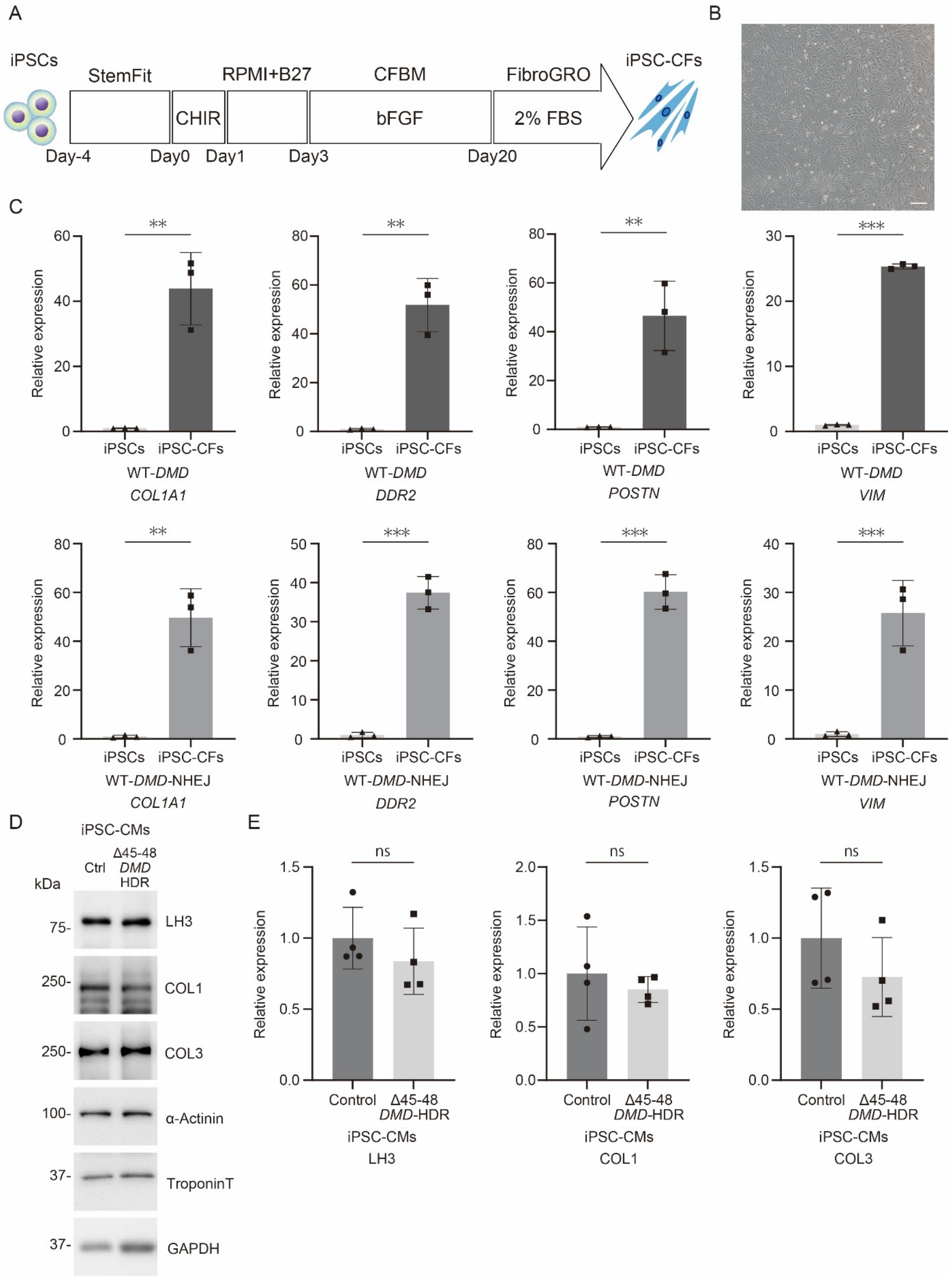

(A) To clarify the functional consequence of LH3 expression in cardiac fibroblasts, WT-*DMD* and WT-*DMD*-NHEJ iPSCs were differentiated to cardiac fibroblast according to the previously established protocol.[8, 9] Quantitative real-time PCR and Western blotting were performed after day 30 of differentiation. **(B)** Representative bright-field image showing differentiated iPSC-CF at day 30 after differentiation. Scale bar; 200 μm. **(C)** The relative expression levels of the marker genes for cardiac fibroblasts (*COL1A1*, *DDR2, POSTN* and *VIM*) normalized by *GAPDH* were measured by quantitative real-time PCR at day 30 (n=3). The relative expression levels normalized to the levels of iPSCs are shown. The expression levels of the marker genes were significantly increased in WT-*DMD* and WT-*DMD*-NHEJ iPSC-CFs compared to WT-*DMD* and WT-*DMD*-NHEJ iPSCs, respectively. ***P*<0.01, ****P*<0.001. **(D)** Whole-cell lysates were extracted from Control and Δ45–48-*DMD*-HDR iPSC-CMs and analyzed by western blotting using the indicated antibodies. **(E)** Relative expression of each protein was normalized to GAPDH and quantitatively analyzed (LH3, n = 4; COL1, n = 4; COL3, n = 4). Relative expression levels normalized to the levels of control iPSC-CMs are shown. ns = not significant.

Supplemental Table. Primer and Probe Sequences and DNA Oligonucleotides Used in This Study.

Oligo DNA		sequence (5'-3')	Application
Hs_DMD_mRNA_F	primer	CCAGCTTGATTTCCAATGGGA	PCR analysis for the deletion site of DMD
Hs_DMD_mRNA_R	primer	GACTGACCACTATTGGAGCCTCT	PCR analysis for the deletion site of DMD
Hs_PLOD3_mRNA_F	primer	GGAGCACTACGGCCAGTG	real-time PCR analysis for the transcript of PLOD3
Hs_PLOD3_mRNA_R	primer	GTGGGCACATTCTCGTAGC	real-time PCR analysis for the transcript of PLOD3
PLOD3_gRNA	sgRNA	GGAAACAGGCTCTCGGTCAT	
Hs_PLOD3_synonymous_F	primer	TATGTGGGACCAATGACCGA	primer for inverse PCR to generate HDR template with synonymous variant for using gRNA
Hs_PLOD3_synonymous_R	primer	CGTCCGCAGCAGCTGCAGCC	primer for inverse PCR to generate HDR template with synonymous variant for using gRNA
Hs_AR_F	primer	TCCAGAATCTGTTCCAGAGCGTGC	PCR analysis for X chromosome inactivation assay
Hs_AR_R	primer	ATGAGGAACAGCAACCTTCACAGC	PCR analysis for X chromosome inactivation assay
Hs_B4GALT1_F	primer	CACCatgaggcttcgggagccgct	cloning from human cDNA
Hs_B4GALT1_R	primer	gctcggtgtcccgatgtccact	cloning from human cDNA
Hs_COL1A1_mRNA_F	primer	GGGATTCCCTGGACCTAAAG	real-time PCR analysis for the transcript of COL1A1

Hs_COL1A1_mRNA_R	primer	GGAACACCTCGCTCTCCA	real-time PCR analysis for the transcript of COL1A1
Hs_DDR2_mRNA_F	primer	AACGAGAGTGCCACCAATGGCT	real-time PCR analysis for the transcript of DDR2
Hs_DDR2_mRNA_R	primer	ACTCACTGGCTTCAGAGCGGAA	real-time PCR analysis for the transcript of DDR2
Hs_POSTN_mRNA_F	primer	CAGCAAACCACCTTCACGGATC	real-time PCR analysis for the transcript of POSTN
Hs_POSTN_mRNA_R	primer	TTAAGGAGGCGCTGAACCATGC	real-time PCR analysis for the transcript of POSTN
Hs_VIM_mRNA_F	primer	AGGCAAAGCAGGAGTCCACTGA	real-time PCR analysis for the transcript of VIM
Hs_VIM_mRNA_R	primer	ATCTGGCGTTCCAGGGACTCAT	real-time PCR analysis for the transcript of VIM

**Custom made primers and probes for ddPCR**		**sequence (5'-3')**	**Application**
Forward primer_DMD Ex 44	primer	TGGGAACATGCTAAATACAA	PCR primer to amplify WT-DMD and Δ45–48-DMD transcripts
Reverse primer_DMD Ex 45	primer	TTTGCCGCTGCCC	PCR primer to amplify WT-DMD transcript
Reverse primer_DMD Ex 49	primer	CCGGTTGTTTAGCTTGAA	PCR primer to amplify Δ45–48-DMD transcript
DMD Ex45-48 WT (HEX)	probe	CATCCTGGAGTTCCTTAAGA	ddPCR probe to detect transcript from WT-DMD allele
DMD Ex45-48 deletion (FAM)	probe	TGCTATTTCAGTTTCCTTAAGA	ddPCR probe to detect transcript from Δ45–48-DMD allele

Supplemental References

1. Shiba M, Higo S, Kondo T, et al. Phenotypic recapitulation and correction of desmoglein-2-deficient cardiomyopathy using human-induced pluripotent stem cell-derived cardiomyocytes. *Hum Mol Genet*. 2021;30:1384-97.
2. Inoue H, Nakamura S, Higo S, et al. Modeling reduced contractility and impaired desmosome assembly due to plakophilin-2 deficiency using isogenic iPS cell-derived cardiomyocytes. *Stem Cell Reports*. 2022;17:337-51.
3. Higo S, Hikoso S, Miyagawa S and Sakata Y. Genome Editing in Human Induced Pluripotent Stem Cells (hiPSCs). *Methods Mol Biol*. 2021;2320:235-45.
4. Burridge PW, Matsa E, Shukla P, et al. Chemically defined generation of human cardiomyocytes. *Nat Methods*. 2014;11:855-60.
5. Li J, Zhang L, Yu L, et al. Circulating re-entrant waves promote maturation of hiPSC-derived cardiomyocytes in self-organized tissue ring. *Commun Biol*. 2020;3:122.
6. Yang KC, Breitbart A, De Lange WJ, et al. Novel Adult-Onset Systolic Cardiomyopathy Due to MYH7 E848G Mutation in Patient-Derived Induced Pluripotent Stem Cells. *J Am Coll Cardiol Basic Trans Science*. 2018;3:728-40.
7. Tulloch NL, Muskheli V, Razumova MV, et al. Growth of engineered human myocardium with mechanical loading and vascular coculture. *Circ Res*. 2011;109:47-59.
8. Zhang J, Tao R, Campbell KF, et al. Functional cardiac fibroblasts derived from human pluripotent stem cells via second heart field progenitors. *Nat Commun*. 2019;10:2238.
9. Zhang H, Tian L, Shen M, et al. Generation of Quiescent Cardiac Fibroblasts From Human Induced Pluripotent Stem Cells for In Vitro Modeling of Cardiac Fibrosis. *Circ Res*. 2019;125:552-66.
10. Suwa Y, Higo S, Nakamoto K, et al. Old-Age Onset Progressive Cardiac Contractile Dysfunction in a Patient with Polycystic Kidney Disease Harboring a PKD1 Frameshift Mutation. *Int Heart J*. 2019;60:220-5.
11. Higasa K, Miyake N, Yoshimura J, et al. Human genetic variation database, a reference database of genetic variations in the Japanese population. *Journal of human genetics*. 2016;61:547-53.
12. Fu W, O'Connor TD, Jun G, et al. Analysis of 6,515 exomes reveals the recent origin of most human protein-coding variants. *Nature*. 2013;493:216-20.
13. Viggiano E, Picillo E, Ergoli M, Cirillo A, Del Gaudio S and Politano L. Skewed X-chromosome inactivation plays a crucial role in the onset of symptoms in carriers of Becker muscular dystrophy. *J Gene Med*. 2017;19.
14. Viggiano E, Picillo E, Cirillo A and Politano L. Comparison of X-chromosome inactivation in Duchenne muscle/myocardium-manifesting carriers, non-manifesting carriers and related daughters. *Clin Genet*. 2013;84:265-70.
15. Komaki R, Hashimoto Y, Mori-Yoshimura M, et al. Severe cardiac involvement with preserved truncated dystrophin expression in Becker muscular dystrophy by +1G>A DMD splice-site

mutation: a case report. *J Hum Genet*. 2020;65:903-9.
16. He R, Li H, Wang L, et al. Engraftment of human induced pluripotent stem cell-derived myogenic progenitors restores dystrophin in mice with duchenne muscular dystrophy. *Biol Res*. 2020;53:22.
17. Yasutake H, Lee JK, Hashimoto A, et al. Decreased YAP activity reduces proliferative ability in human induced pluripotent stem cell of duchenne muscular dystrophy derived cardiomyocytes. *Sci Rep*. 2021;11:10351.
